# Global soil moisture data fusion by Triple Collocation Analysis from 2011 to 2018

**DOI:** 10.1038/s41597-022-01772-x

**Published:** 2022-11-11

**Authors:** Qiuxia Xie, Li Jia, Massimo Menenti, Guangcheng Hu

**Affiliations:** 1 School of Surveying and Geo-Informatics, Shandong Jianzhu University, Jinan, 250101 China; 2 State Key Laboratory of Remote Sensing Science, Aerospace Information Research Institute, Chinese Academy of Sciences, Beijing, 100101 China; 3 Delft University of Technology, Delft, The Netherlands

**Keywords:** Hydrology, Hydrogeology

## Abstract

Surface Soil Moisture (SSM) information is needed for agricultural water resource management, hydrology and climate analysis applications. Temporal and spatial sampling by the space-borne instruments designed to retrieve SSM is, however, limited by the orbit and sensors of the satellites. We produced a Global Daily-scale Soil Moisture Fusion Dataset (GDSMFD) with 25 km spatial resolution (2011~2018) by applying the Triple Collocation Analysis (TCA) and Linear Weight Fusion (LWF) methods. Using five metrics, the GDSMFD was evaluated against *in-situ* soil moisture measurements from ten ground observation networks and compared with the prefusion SSM products. Results indicated that the GDSMFD was consistent with *in-situ* soil moisture measurements, the minimum of root mean square error values of GDSMFD was only 0.036 cm^3^/cm^3^. Moreover, the GDSMFD had a good global coverage with mean Global Coverage Fraction (GCF) of 0.672 and the maximum GCF of 0.837. GDSMFD performed well in accuracy and global coverage fraction, making it valuable in applications to the global climate change monitoring, drought monitoring and hydrological monitoring.

## Abstract (summary)

**Table:** 

Measurement(s)	surface soil moisture
Technology Type(s)	microwave satellite imaging
Sample Characteristic - Environment	planetary surface
Sample Characteristic - Location	Global

## Background & Summary

Information on Surface Soil Moisture (SSM) plays a key role for many practical applications, such as agricultural water management, global weather forecasts, hydrology and natural disasters monitoring. SSM is also an important variable in water and energy exchanges at the atmosphere/land-surface interface^1,2^. Since 1970s active and passive microwave remote sensing instruments and algorithms to retrieve near-surface soil moisture (top 5 cm depth), have improved to make it possible to monitor global SSM with sufficient accuracy and spatio-temporal resolution^3–5^. Active sensors mainly include the Advanced Scatterometer (ASCAT) onboard the Meteorological Operational Satellite-A/B (MetOp-A/B) (2007-present) and the radar onboard Soil Moisture Active and Passive mission (SMAP) (2015-present). Passive sensors mainly include the Tropical Rainfall Measuring Mission’s (TRMM) Microwave Imager (TMI) (1997–2015), the Advanced Microwave Scanning Radiometer-Earth Observing System (AMSR-E) onboard Aqua satellite (2002~2011), the Coriolis satellite WindSat (2003–2012), the Soil Moisture and Ocean Salinity (SMOS) (2010-present)^6^, the MicroWave Radiation Imager (MWRI) of FenYun-3B (FY3-B) (2011–2019) and FenYun-3C (FY3-C) (2014-present) satellites, the Advanced Microwave Scanning Radiometer-2 (AMSR-2) of Global Change Observation Mission 1^st^ - Water “SHIZUKU” (GCOM-W1) (2012-present) and the radiometer onboard SMAP (2015-present).

Based on observations of active/passive microwave remote sensing instruments, many global SSM products such as AMSR-E, AMSR-2, ASCAT, SMOS, SMAP and the European Space Agency-Climate Change Initiative (ESA-CCI) were produced and evaluated using *in-situ* soil moisture measurements and airborne observations^3,7–9^. Particularly, the *in-situ* soil moisture measurements from the International Soil Moisture Network (ISMN) and simulated SSM data by land surface models were used for this purpose. However, previous studies found that no global active/passive SSM products performed superior to other products everywhere and performance seemed to depend on land cover^10^. In addition, there are gaps in the global SSM products due to incomplete coverage of satellite orbits and retrieval errors. These shortcomings limited these soil moisture data for long-time applications in, e.g., agricultural drought monitoring and water resources management.

Data fusion to merge different global SSM data product seems the most effective solution to overcome the shortcomings identified above^11^. Data fusion can integrate multiple remote sensing data sets and knowledge into a better data product^12^. Examples of such algorithms are the Linear Weight Fusion (LWF), non-Linear WF, multiple linear regression, artificial neural network, Copula-based data fusion and least square method-based image fusion^13–16^. The LWF algorithm, combining data sets linearly based on the weight values of components to be fused, is the most practical and well understood fusion algorithm^17^. The key to the LWF algorithm is to calculate the weight values of the different components on the basis of the correlations or errors of different components. At present, Triple Collocation Analysis (TCA) method is one of the most popular methods for estimating random errors in soil moisture retrievals without reliable ground observations^18^. TCA was first proposed to estimate ocean wind speed errors and to correct ocean wind speed^19^, and subsequently used to estimate uncertainties in remote sensing retrievals of, e.g., soil moisture and leaf area index^20^. Therefore, it will be potential by merging of existential soil moisture based on the combing algorithm of TCA and LWF methods for improvement of the global SSM product with 25 km SGR.

**Fig. 1:**
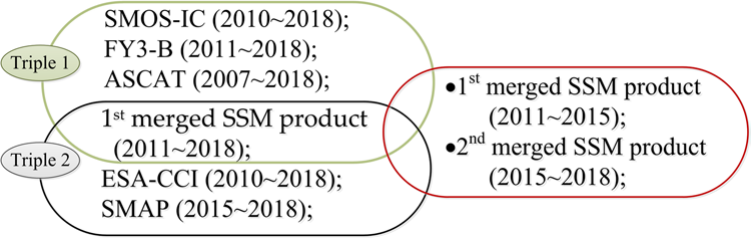
Overview of the two-triplet merging approach from global-scale original SSM products (SMOS, FY3-B, ASCAT, ESA-CCI and SMAP) to final merged SSM products (1^st^ and 2^nd^ merged SSM products).

Here, we present a novel global daily-scale SSM product (i.e., GDSMFD) with 25 km SGR in good accuracy and global coverage using the easiest and well understood LWF method to merging SMOS, FY3-B, ASCAT, ESA-CCI and SMAP SSM products (i.e. SMOS SSM retrieved using L-MEB algorithm, FY3-B SSM retrieved using the soil moisture retrieval algorithm based on Qp roughness model, ASCAT SSM retrieved using the SWI algorithm based on change monitoring method, ESA-CCI SSM retrieved using the data fusion procedure and SMAP SSM retrieved using SCA-V algorithm). We calculated the pixel-wised weight values of the SM values from SMOS, FY3-B, ASCAT, SMAP SSM and ESA-CCI SSM products based on TCA. We applied a strategy that first SMOS, FY3-B and ASCAT SSM products from 2011 to 2018 were merged using the TCA LWF method to produce the 1^st^ merged SSM product which is further merged with SMAP-SSM and ESA-CCI-SM products to obtain the 2^nd^ merged SSM product for 2015~2018 (SMAP-SSM available from 2015) by using the same TCA LWF method (Fig. 1). The above two merged SM datasets composed our time series of fusion SM data between 2011–2018.

## Methods

### Global soil moisture fusion data preparation

Five satellite surface soil moisture products including three passive microwave soil moisture products (i.e., SMOS-IC, FY3-B and SMAP), one active microwave soil moisture products (i.e., ASCAT) and one merged soil moisture product (i.e., ESA-CCI) were used in this study to generate the merged dataset (Table 1), the details are described below.

**Table 1:** Information on five active and passive microwave SSM products used in this study.

Products	Versions	Period	Sensor types	Used Band	SGR	Unit	Algorithms	Main reference
SMOS-IC	L3	2010~2018	Passive	1.41 GHz	25 km	cm^3^/cm^3^	L-MEB	Wigneron *et al*.^23^
FY3-B	L2	2011~2018	Passive	10.7 GHz	25 km	cm^3^/cm^3^	Qp	Shi *et al*.^27^
ASCAT	V3.0	2007~2018	Active	5.3 GHz	0.1°	%	TU-Wien	Wagner *et al*.^29^
SMAP	L3	2015~2018	Passive	1.41 GHz	9 km	cm^3^/cm^3^	SCA-V	Jackson^37^
ESA-CCI	V4.5	1978~2018	/	/	0.25°	cm^3^/cm^3^	Merged	Gruber *et al*.^20^

SMOS satellite launched in November 2009 by ESA carries the Microwave Interferometric Radiometer with Aperture Synthesis (MIRAS), a synthetic aperture microwave radiometer to observe the dual-polarized, multi-angular brightness temperature in L band (1.4 GHz) and designed to retrieve global SSM at 3~5 cm soil depth with high accuracy^21^. In this study, we used the new global SMOS-IC L3 SSM product with 25 km EASE 2.0 SGR generated by the Institut National de la Recherche Agronomique-Centre d’Etudes Spatiales de la Biosphère (INRA-CESBIO) (http://bec.icm.csic.es/data/data-access/)^22,23^. The SMOS-IC L3 SSM retrieval algorithm was based on the L-MEB to make soil moisture retrieval as independent as possible from auxiliary data such as the Leaf Area Index (LAI) and Normalized Difference Vegetation Index (NDVI)^23,24^. The SMOS-IC L3 SSM algorithm considered pixels as homogeneous avoiding uncertainties related to auxiliary data sets that were used to characterize the pixel heterogeneity in e.g. forest areas, which different from SMOS L2 SSM^25^. Compared to SMOS L2 SSM products, the SMOS-IC L3 SSM product is more robust in reducing the effect of vegetation and surface roughness^23^.

FY3-B launched on November 5, 2010, was the second polar-orbiting satellite of the FY3 meteorological satellite series and carried the MWRI operating at 5 frequencies, i.e., 10.65, 18.7, 23.8, 36.5 and 89 GHz, and Horizontal (H) and Vertical (V) polarization. MWRI is one of the eleven instruments onboard the FY3-B satellite and measures the exitance of the earth-atmosphere system^26^. In this study, we used the daily FY3-B SSM product with 25 km SGR and a nominal sensing soil depth of 2 cm and applying a retrieval algorithm including an improved Qp surface roughness model developed by Shi *et al*.^27^. This product was provided by the National Satellite Meteorological Center (NSMC) of China (http://satellite.nsmc.org.cn/PortalSite/Default.aspx). The improved Qp model reduces the effect of surface roughness on the soil moisture retrieval. An empirical vegetation opacity model using Vegetation Water Content (VWC), assumed to depend only on LAI was applied to correct the effect of vegetation on FY3-B/MWRI brightness temperature observations^27^.

ASCAT sensors onboard the MetOp-A and MetOp-B launched by ESA since 2006 are real aperture radar sensors operating in C band (5.255 GHz) using vertical polarization antennas to measure global radar backscatter at 25 km SGR with 1~3 days revisit time. In this study, we used the daily ASCAT SSM product with 10 km SGR released by the Copernicus Global Land Service (CGLS) based on the SWI algorithm developed by the Vienna University of Technology (https://land.copernicus.eu/global/products/swi)^28^. The SWI algorithm is based on a simple soil moisture infiltration model proposed by Wagner *et al*.^29^ to estimate profile soil moisture from ASCAT backscatter observations^29^. ASCAT product has eight SWI layers according to different *T* values (i.e., 1, 5, 10, 15, 20, 40, 60 and 100). In the SWI algorithm, the *T* value is a function (*T* = *L*/*C)* of *L* (the depth of the soil layer) and *C* (the area-representative pseudo diffusivity constant) parameters, which means a high *T* describes a deeper soil layer if the soil water diffusivity is constant. Therefore, this study used the SWI data of the first layer (*T* = 1) so that each pixel value represents the degree of saturation (%) with a nominal sensing depth of 1~5 cm.

ESA-CCI V4.5 SSM product was developed by ESA in 2010 using the TCA-LWF algorithm applied to either backscatter coefficient or brightness temperature retrieved with three active sensors, i.e., European Remote Sensing-1/2 (ERS-1/2), MetOp-A/ASCAT, and MetOp-B/ASCAT, and seven passive sensors, i.e. Nimbus-7’s Scanning Multichannel Microwave Radiometer (SMMR), Special Sensor Microwave-Imager (SSM/I), TRMM, AMSR-E, AMSR-2, WindSat, and SMOS^15,20,30–33^. Currently, it is one of the most widely used SSM data products in the world (https://esa-soilmoisture-cci.org/). The three soil moisture data products based on the measurements by active instruments were retrieved using the same soil moisture retrieval algorithm, i.e., the change detection method developed by the Vienna University of Technology (TU-Wien) WARP v5.5, from backscatter coefficient observed by ERS-1/2, MetOp-A/ASCAT, and MetOp-B/ASCAT, then were merged using the TCA-LWF algorithm. The seven SSM data products based on the measurements by microwave radiometers were retrieved using the same soil moisture retrieval algorithm, i.e., the LPRM developed by National Aeronautics and Space Administration (NASA), from brightness temperature observed by SMMR, SSM/I, TRMM, AMSR-E, AMSR2, WindSat and SMOS, then were merged using the TCA-based LWF algorithm. Finally, the active and passive merged data products were blended into one final dataset. In this study, we merged the final combined dataset with SMOS, ASCAT, FY3-B and SMAP SSM products.

SMAP was launched by NASA in January 2015 and includes a L-band (1.26 GHz) radar and a L-band (1.41 GHz) radiometer. This unique near-polar sun-synchronous satellite monitors global SSM in the top 5 cm soil and freeze/thaw state with higher accuracy, coverage and resolution than previous microwave systems^34^. Currently, there are three SMAP SSM products with 3 km, 9 km, and 36 km SGR available. The L-band radar with higher SGR (1~3 km) stopped working after 11 weeks of operation, however. Therefore, only SMAP SSM products with 9 km and 36 km SGR are available from 2015 to the present. In this study we used the enhanced L3 SMAP SSM product assuming a 5 cm soil sensing depth with 9 km EASE 2.0 SGR released by the Nation Snow & Ice Data Center (NSIDC) (http://nsidc.org/data/)^35^. This product is a daily composite of the enhanced SMAP L2 half-orbit SSM data with 9 km SGR, retrieved from SMAP L1 interpolated brightness temperature data with 36 km SGR using the SCA-V algorithm^36–38^.

### Soil moisture validation data preparation

**Fig. 2:**
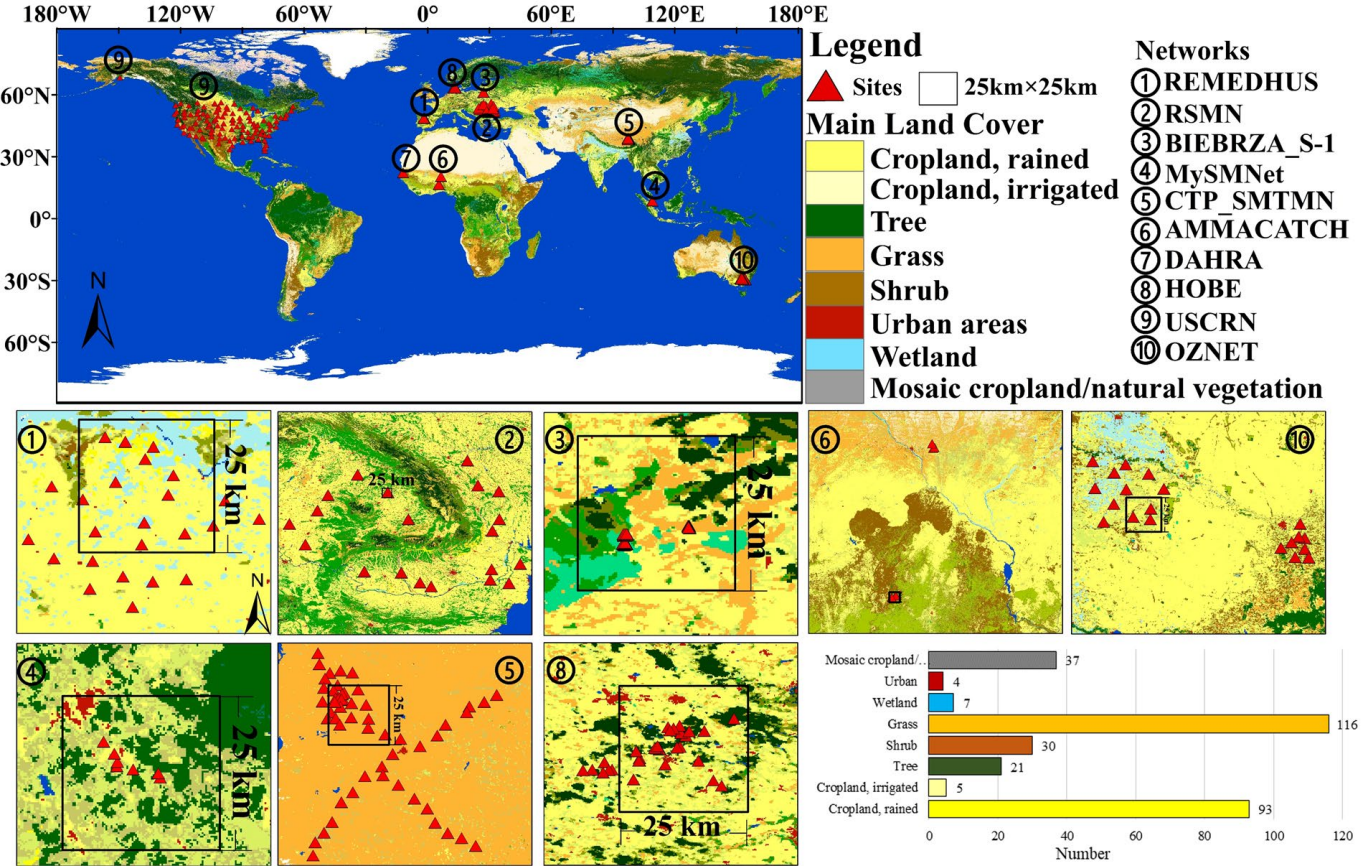
Distribution of *in-situ* soil moisture measurement stations and LC characteristics of the networks listed in Table 2.

*In-situ* soil moisture measurements collected at a total 311 sites of the International Soil Moisture Network (ISMN) (https://ismn.geo.tuwien.ac.at/en/), specifically 10 contributing networks, i.e. CTP_SMTMN, RSMN, AMMACATCH, DAHRA, BIEBRZA_S-1, MySMNet, REMEDHUS, HOBE, USCRN and OZNET, with dense sites in 9 countries, i.e. Poland, Spain, China, Malaysia, Romania, Benin, Niger, Mali, Senegal, USA, Australia and Denmark were used to verify the TCA assumptions and evaluate SMOS, FY3-B, ASCAT, ESA-CCI, SMAP satellite SSM products and the merged SSM product developed by this study using the TCA-LWF algorithm (Table 2 and Fig. 2)^39–49^. These 311 sites are part of 10 networks distributed in five continents: Asia, Europe, Africa, North America and Australia. Particularly, in the CTP_SMTMN in China, there are about 20 sites in a pixel of the SSM product with 25 km SGR. The mean value of the *in-situ* soil moisture data in a pixel of the SSM products was considered as the true value of soil moisture. Also, we used the ESA-CCI L4 global 2015 Land Cover (LC) map with 300 m SGR to identify the land cover type of each *in-situ* soil moisture measurement site (http://www.esa-landcover-cci.org/). The soil moisture measurements are obtained at different soil depths, i.e., 5 cm, 10 cm, 20 cm and 40 cm. In this study, we used the *in-situ* soil moisture measurements at 5 cm soil depth, approximately consistent with the nominal sensing depth of microwave SSM L-band instruments.

**Table 2:** Information on *in-situ* soil moisture measurements used in this study (*N*: the number of sites).

Networks	Countries	N	Instruments	Obs. Depth	Period (dd/mm/yy)	References
CTP_SMTMN	China	57	5TM/EC-TM	5 cm	01/08/2010~19/09/2016	Yang *et al*.^40^
RSMN	Romania	20	5TM (0~1)	5 cm	09/04/2014~15/05/2020	Sandric *et al*.^41^
AMMACATCH	Benin, Niger, Mali	7	CS616	5 cm	01/01/2006~31/12/2018	Pellarin *et al*.^42^
DAHRA	Senegal	1	Theta Probe ML2X	5 cm	04/07/2002~01/01/2016	Tagesson *et al*.^43^
BIEBRZA_S-1	Poland	28	GS-3(0~1)	5 cm	23/04/2015~01/12/2018	Musial *et al*.^44^
MySMNet	Malaysia	7	Water Scout SM100	5 cm	31/05/2014~31/12/2015	Kang *et al*.^45^
REMEDHUS	Spain	24	Stevens Hydra Probe	5 cm	15/03/2005~01/01/2020	Martínez-Fernández *et al*.^46^
HOBE	Denmark	32	Decagon 5TE	5 cm	08/09/2009~13/03/2019	Bircher *et al*.^47^
USCRN	USA	115	Stevens Hydra probe II Sdi-12	5 cm	15/11/2000~26/10/2020	Bell *et al*.^48^
OZNET	Australia	20	Stevens Hydra Probe	5 cm	12/09/2001~27/08/2018	Young *et al*.^49^

We identified the land cover type at the sites described above by applying the ESA-CCI L4 2015 global LC map with 300 m SGR (Fig. 2). The land cover types of ESA-CCI LC data were defined using the United Nations-Land Cover Classification System (UN-LCCS) which considers 40 classes. The 311 ISMN sites were distributed mainly in 8 classes (Fig. 2), with most sites being located in grassland and cropland areas, followed by shrub, tree and mosaic cropland/natural vegetation (tree, shrub and grass) (37). In the REMEDHUS network of Spain, there were 24 sites, among which 16 sites were located in herbaceous cover area. In the RSMN network of Romania, 20 sites were distributed mainly in the rainfed cropland and herbaceous cover area. In the BIEBRZA_S-1 network of Poland, 28 sites were mainly in rainfed cropland and grassland, others in shrubland or herbaceous cover, flooding, fresh/saline/brackish water area. In the CTP_SMTMN network, the land cover of 56 out of 57 sites was grassland. In the MySMNet network of Malaysia, 4 out of 7 sites were in the mosaic nature vegetation (tree, shrub, herbaceous >50%)/ cropland (<50%). In DAHRA and AMMA-CATCH networks of Africa, out of 8 sites, 2 sites were in the broadleaf deciduous tree (15~40%), 2 sites in shrubland and 2 sites in grassland. In HOBE 11 out of 32 sites are located in raining cropland, while the remaining ones were concentrated in tree and mosaic cropland/natural vegetation in the USCRN, 45 out of 115 were in grassland and 24 sites were in shrub land. In OZNET, 8 out of 20 were in rained cropland, 4 in shrub land and 5 in mixed vegetation. Overall, 116 of the 311 ISMN sites were in grassland, 93 in cropland, 30 in shrub land, 21 in tree and the remaining ones were in mosaic cropland/natural vegetation (tree, shrub and grass).

### Triple collocation analysis

At present, the TCA method is one of the most widely used evaluation methods for satellite SSM products in the absence of *in-situ* soil moisture measurement data^50^. The evaluation of errors in satellite SSM data products using TCA is based on three assumptions: a) soil moisture retrievals are linearly related to the true soil moisture value; b) the errors on each SSM retrieval are uncorrelated with the true soil moisture; and c) errors within each selected triplet of SSM retrievals are uncorrelated with each other^20^.

The retrieved soil moisture (SM, cm^3^/cm^3^) in each pixel can then be expressed as a linear relationship between the true soil moisture value (*t*, cm^3^/cm^3^) and random error (*ε*) as follows^50^:

1 $${\rm{SM}}=\alpha +\beta \ast t+\varepsilon $$

Where *α* and *β* are the coefficients.

The covariance (${\rm{Cov}}\left(S{M}_{i{\prime} }S{M}_{j}\right)$) of two SSM products (*SM*_*i*_ and *SM*_*j*_) is then:

2 $${\rm{Cov}}\left({{\rm{SM}}}_{{\rm{i}}},{{\rm{SM}}}_{{\rm{j}}}\right)={\beta }_{i}{\beta }_{j}{\sigma }_{t}^{2}+{\beta }_{i}{\rm{Cov}}\left(t,{\varepsilon }_{j}\right)+{\beta }_{j}{\rm{Cov}}\left(t,{\varepsilon }_{i}\right)+{\rm{Cov}}\left({\varepsilon }_{i},{\varepsilon }_{j}\right)$$

3 $${Q}_{ij}=\left[\begin{array}{ccc}{Q}_{11} & {Q}_{12} & {Q}_{13}\\ {Q}_{21} & {Q}_{22} & {Q}_{23}\\ {Q}_{31} & {Q}_{32} & {Q}_{33}\end{array}\right],\left\{\begin{array}{c}i=1,2,3\\ j=1,2,3\end{array}\right.$$

Where ${\sigma }_{t}^{2}$ is the variance of true soil moisture value (*t*, cm^3^/cm^3^); Cov(*SM*_*i*′_ *SM*_*j*_) is the covariance matrix of paired data products, with three pairs in each triplet when applying TCA (Eq. 3) (Table 3).

**Table 3:** Pair strategy of SMOS, FY3-B, ASCAT, SMAP and ESA-CCI for covariance estimation in the 1^st^ and 2^nd^ merging procedures.

Steps	Pairs	Results
Triplet 1	SMOS, FY3-B	1^st^ merged SSM product
	SMOS, ASCAT	
	FY3-B, ASCAT	
Triplet 2	1^st^ merged SSM, ESA-CCI	2^nd^ merged SSM product
	1^st^ merged SSM, SMAP	
	ESA-CCI, SMAP	

In each triplet, three soil moisture datasets were retrieved using different algorithms and from different data sources. Accordingly, it was assumed that zero error cross-correlation between each pair of soil moisture data sets was nihil, i.e., Cov(*ε*_*i*_, *ε*_*j*_) = *0, i ≠ j*, and that the error - on “true” soil moisture and the error on each soil moisture dataset i.e., Cov(*t*, *ε*_*i*_) = 0 were orthogonal. Based on these assumptions, Eq. 2 can be rewritten as:

4 $${Q}_{ij}={\rm{Cov}}({{\rm{SM}}}_{{\rm{i}}},{{\rm{SM}}}_{{\rm{j}}})=\left\{\begin{array}{l}{\beta }_{i}{\beta }_{j}{\sigma }_{t}^{2},i\ne j\\ {\beta }_{i}{\beta }_{j}{\sigma }_{t}^{2}+{\sigma }_{\varepsilon i}^{2},i=j\end{array},\left\{\begin{array}{c}i=1,2,3\\ j=1,2,3\end{array}\right.\right.$$

Since there are six unique terms (*Q*_11_*, Q*_12_ = *Q*_21_*, Q*_13_ = *Q*_31_*, Q*_22_*, Q*_23_ = *Q*_32_*, Q*_33_) in the 3 × 3 covariance matrix (Eq. 3), we can obtain six equations but seven unknowns (${\beta }_{1},{\beta }_{2},{\beta }_{3},{\sigma }_{\varepsilon 1}^{2},{\sigma }_{\varepsilon 2}^{2},{\sigma }_{\varepsilon 3}^{2},{\sigma }_{t}^{2}$). Therefore, there is no unique solution. A new variable *θ*_*i*_ = *β*_*i*_*σ*_*t*_ is introduced in Eq. 4, which can be rewritten as:

5 $${Q}_{ij}=\left\{\begin{array}{l}{\theta }_{i}{\theta }_{j},i\ne j\\ {\theta }_{i}^{2}+{\sigma }_{\varepsilon i}^{2},i=j\end{array},\left\{\begin{array}{c}i=1,2,3\\ j=1,2,3\end{array}\right.\right.$$

In Eq. 5, there are six equations and six unknowns. Therefore, a unique value for each unknown can be calculated. Finally, the error variance values on the three independent SSM products in each triplet (Table 3) (${\sigma }_{\varepsilon 1},{\sigma }_{\varepsilon 2},{\sigma }_{\varepsilon 3}$) can be estimated as:

6 $$\left\{\begin{array}{c}{\sigma }_{\varepsilon 1}^{2}\\ {\sigma }_{\varepsilon 2}^{2}\\ {\sigma }_{\varepsilon 3}^{2}\end{array}=\left[\begin{array}{c}{Q}_{11}-\frac{{Q}_{12}{Q}_{13}}{{Q}_{23}}\\ {Q}_{22}-\frac{{Q}_{12}{Q}_{23}}{{Q}_{13}}\\ {Q}_{33}-\frac{{Q}_{23}{Q}_{13}}{{Q}_{12}}\end{array}\right]\right.$$

### The linear weight fusion (LWF) method

The key in the LWF method is to estimate the weight value of each pixel of each SSM product. When the soil moisture values of three SSM products in 1^st^ or 2^nd^ step of the merging procedure are available, the weight values of three SSM products (*w*_1_, *w*_2_, *w*_3_) can be calculated by using the estimated error variance values on three SSM products from Eq. 6 as:

7 $$\left\{\begin{array}{c}{w}_{1}\\ {w}_{2}\\ {w}_{3}\end{array}\right.=\left\{\begin{array}{c}\frac{1/{\sigma }_{\varepsilon 1}^{2}}{1/{\sigma }_{\varepsilon 1}^{2}+1/{\sigma }_{\varepsilon 2}^{2}+1/{\sigma }_{\varepsilon 3}^{2}}\\ \frac{1/{\sigma }_{\varepsilon 2}^{2}}{1/{\sigma }_{\varepsilon 1}^{2}+1/{\sigma }_{\varepsilon 2}^{2}+1/{\sigma }_{\varepsilon 3}^{2}}\\ \frac{1/{\sigma }_{\varepsilon 3}^{2}}{1/{\sigma }_{\varepsilon 1}^{2}+1/{\sigma }_{\varepsilon 2}^{2}+1/{\sigma }_{\varepsilon 3}^{2}}\end{array}\right.,{w}_{1}+{w}_{2}+{w}_{3}=1$$

When the soil moisture values of only two of three SSM products in 1^st^ or 2^nd^ step of the merging procedure are available, the weight values of two of three SSM products are re-estimated by using the estimated error variance values on two of the three SSM products from Eq. 6 as:

8 $$\left\{\begin{array}{c}{w}_{1}\\ {w}_{2}\end{array}\right.=\left\{\begin{array}{c}\frac{\frac{1}{{\sigma }_{\varepsilon 1}^{2}}}{\frac{1}{{\sigma }_{\varepsilon 1}^{2}}+\frac{1}{{\sigma }_{\varepsilon 2}^{2}}}\\ \frac{\frac{1}{{\sigma }_{\varepsilon 2}^{2}}}{\frac{1}{{\sigma }_{\varepsilon 1}^{2}}+\frac{1}{{\sigma }_{\varepsilon 2}^{2}}}\end{array}\right.,{w}_{1}+{w}_{2}=1\;{\rm{or}}\;\left\{\begin{array}{c}{w}_{1}\\ {w}_{3}\end{array}\right.=\left\{\begin{array}{c}\frac{\frac{1}{{\sigma }_{\varepsilon 1}^{2}}}{\frac{1}{{\sigma }_{\varepsilon 1}^{2}}+\frac{1}{{\sigma }_{\varepsilon 3}^{2}}}\\ \frac{\frac{1}{{\sigma }_{\varepsilon 3}^{2}}}{\frac{1}{{\sigma }_{\varepsilon 1}^{2}}+\frac{1}{{\sigma }_{\varepsilon 3}^{2}}}\end{array}\right.,{w}_{1}+{w}_{3}=1\;{\rm{or}}\;\left\{\begin{array}{c}{w}_{2}\\ {w}_{3}\end{array}\right.=\left\{\begin{array}{c}\frac{\frac{1}{{\sigma }_{\varepsilon 2}^{2}}}{\frac{1}{{\sigma }_{\varepsilon 2}^{2}}+\frac{1}{{\sigma }_{\varepsilon 3}^{2}}}\\ \frac{\frac{1}{{\sigma }_{\varepsilon 3}^{2}}}{\frac{1}{{\sigma }_{\varepsilon 2}^{2}}+\frac{1}{{\sigma }_{\varepsilon 3}^{2}}}\end{array}\right.,{w}_{2}+{w}_{3}=1$$

When only one soil moisture values of the three SSM data products in 1^st^ step or 2^nd^ step is available, the weight value of one of three SSM products is equal to 1 and weight = 0 for the two remaining SSM products. In this study, the scheme to estimate the weight values of three SSM products in 1^st^ and 2^nd^ steps is summarized in Table 4. When the temporal coverage of one or more data sets in the triplet is lower, TCA results are often considered unreliable. Therefore, the minimum number of valid samples in each pixel for each triplet in the time-series was set at 100^51^.

**Table 4:** Scheme to assign the weight values of three SSM products in 1^st^ and 2^nd^ steps (×: soil moisture value of SSM product is not available; О: soil moisture value of SSM product is available).

Merge Steps	SMOS	FY3-B	ASCAT	Weight values	Result
Triplet 1	×	×	×	*w*_1_ = *w*_2_ = *w*_3_ = 0	1^st^ merged SSM product
	О	×	×	*w*_1_ = 1, *w*_2_ = *w*_3_ = 0	
	×	О	×	*w*_2_ = 1, *w*_1_ = *w*_3_ = 0	
	×	×	О	*w*_3_ = 1, *w*_1_ = *w*_2_ = 0	
	О	О	×	*w*_1_ and *w*_2_ by Eq. 8, *w*_3_ = 0	
	×	О	О	*w*_2_ and *w*_3_ by Eq. 8, *w*_1_ = 0	
	О	×	О	*w*_1_ and *w*_3_ by Eq. 8, *w*_2_ = 0	
	О	О	О	*w*_1_, *w*_2_ and *w*_3_ by Eq. 7	
	**1**^**st**^ **merged SSM**	**ESA-CCI**	**SMAP**	**Weight values**	**Result**
Triplet 2	×	×	×	*w*_1_ = *w*_2_ = *w*_3_ = 0	2^nd^ merged SSM product
	О	×	×	*w*_1_ = 1, *w*_2_ = *w*_3_ = 0	
	×	О	×	*w*_2_ = 1, *w*_1_ = *w*_3_ = 0	
	×	×	О	*w*_3_ = 1, *w*_1_ = *w*_2_ = 0	
	О	О	×	*w*_1_ and *w*_2_ by Eq. 8, *w*_3_ = 0	
	×	О	О	*w*_2_ and *w*_3_ by Eq. 8, *w*_1_ = 0	
	О	×	О	*w*_1_ and *w*_3_ by Eq. 8, *w*_2_ = 0	
	О	О	О	*w*_1_, *w*_2_ and*w*_3_ by Eq. 7	

The TCA–LWF method was used to merge the SSM data products in each triplet (Table 3). This method is simple and feasible, and the error attributes of every SSM product that are estimated using TCA algorithm are considered. By using the calculated weight values of three SSM products (*w*_1_, *w*_2_, *w*_3_) (Table 4), three SSM products (SM_1_, SM_2_, SM_3_) can be merged by calculating their weighted average as:

9 $$S{M}_{f}={w}_{1}\ast {{\rm{SM}}}_{1}+{w}_{2}\ast {{\rm{SM}}}_{2}+{w}_{3}\ast {{\rm{SM}}}_{3}$$

**Fig. 3:**
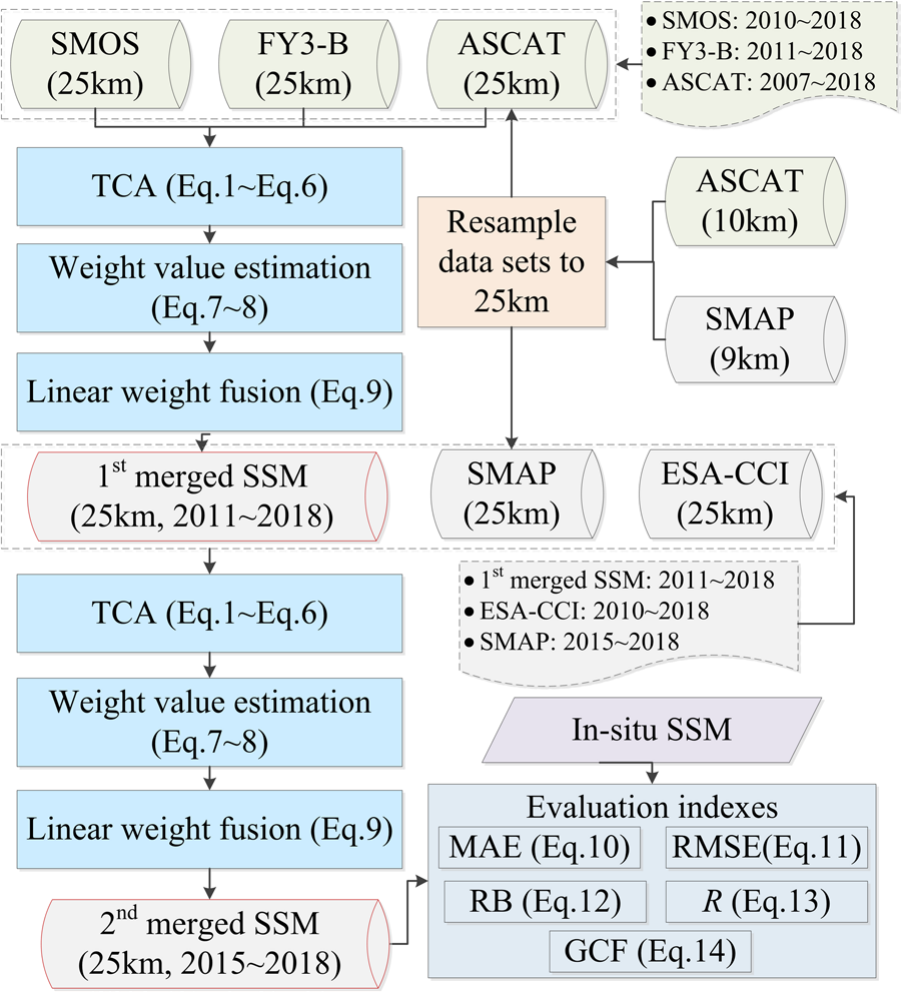
Flowchart of work-flow to merge the SMOS, FY3-B, ASCAT, ESA-CCI, and SMAP SSM products using TCA method.

Based on preliminary assessment analysis of the satellite SSM products, the ESA-CCI SSM product has better performance in accuracy and effective coverage area than AMSR-2, AMSR-E, WindSat and TRMM/TMI SSM products^3,9,10,26,52^. Therefore, this was the reason that the ESA-CCI SSM product was selected and used in the 2^nd^ triplet to obtain the 2^nd^ merged (final) SSM. To limit the complexity of the merging procedure, i.e., the number of unknowns, we split it into two steps, each dealing with three SSM data products to get finally a single merged product from the SMOS, FY3-B, ASCAT, ESA-CCI, and SMAP SSM products. In the first step, the SMOS, FY3-B, and ASCAT SSM products are merged by using a LWF algorithm to obtain the 1^st^ merged SSM product from 2011~2018. Second, the latter is merged with the ESA-CCI and SMAP SSM products by using the same algorithm i.e., the LWF algorithm from 2015~2018. The specific flow chart of SSM product fusion using the LWF algorithm is shown in Fig. 3.

Prior to the fusion of the SMOS, FY3-B, ASCAT, ESA-CCI and SMAP datasets using TCA-based LWF algorithm, unit conversion, abnormal pixel removal, masking and resampling were applied to these five SSM products. All SSM products, i.e., SMOS, FY3-B, ASCAT, ESA-CCI, and SMAP SSM, were resampled to a consistent SGR i.e., 25 km by interpolation of adjacent pixels. In addition, the soil moisture units of SMOS, FY3-B, ESA-CCI and SMAP are the same, i.e., volumetric soil water content (cm^3^/cm^3^), but ASCAT SSM is expressed as saturation (%). To keep consistent units. i.e. volumetric soil water content, cm^3^/cm^3^, we used the global porosity data with 1-degree SGR, released by the Goddard Earth Sciences Data and Information Services Center (GES DISC), were used to convert the ASCAT SWI product to volumetric soil water content (cm^3^/cm^3^)^53^. After resampling and unit conversion, the range of ASCAT SSM was 0–1.0 cm^3^/cm^3^, i.e., the same as SMAP. For SMOS product, the abnormal SMOS SSM values >1.0 or <0 cm^3^/cm^3^, caused by the L-MEB retrieval algorithm, were removed, so that the range of SMOS SSM was 0~1.0 cm^3^/cm^3^, i.e., the same as ASCAT and SMAP. The ranges of ESA-CCI and FY3-B SSM values were 0~0.6 cm^3^/cm^3^ and 0~0.5 cm^3^/cm^3^. Moreover, in order to keep soil moisture information from SMOS, FY3-B, ASCAT, ESA-CCI and SMAP SSM products as possible, the rescaling and masking processes were not implemented for these five original SSM products, but only for the final 1^st^ merged and 2^nd^ merged SSM products. The SSM values >0.6 cm^3^/cm^3^ in the 1^st^ merged and 2^nd^ merged SSM products were replaced using the saturated soil water content data released by the Land-Atmosphere Interaction Research Group at Sun Yat-sen University. The water bodies and permanent snow and ice according to the ESA-CCI L4 global LC map were masked in the final 1^st^ merged and 2^nd^ merged SSM products.

### Metrics for evaluation

In this study, the Mean Absolute Error (MAE, cm^3^/cm^3^), Root Mean Square Error (RMSE, cm^3^/cm^3^), Relative Bias (RB), correlation coefficient (*R*) and Global Coverage Fraction (GCF) were used to compare and assess the 1^st^ and 2^nd^ merged SSM products, as well as the SMOS, FY3-B, ASCAT, ESA-CCI, SMAP:

10 $${\rm{MAE}}=\frac{{\sum }_{D=1}^{N}\left({{\rm{SM}}}_{D}^{E}-{{\rm{SM}}}_{D}^{O}\right)}{N}$$

11 $${\rm{RMSE}}=\sqrt{\frac{{\sum }_{D=1}^{N}{\left({{\rm{SM}}}_{D}^{E}-{{\rm{SM}}}_{D}^{O}\right)}^{2}}{N}}$$

12 $${\rm{RB}}=\frac{{\sum }_{D=1}^{N}\left({{\rm{SM}}}_{D}^{{\rm{E}}}-{{\rm{SM}}}_{D}^{{\rm{O}}}\right)}{{\sum }_{D=1}^{N}{{\rm{SM}}}_{D}^{{\rm{O}}}}$$

13 $$R=\frac{{\rm{cov}}({{\rm{SM}}}_{D}^{E},{{\rm{SM}}}_{D}^{O})}{{\sigma }_{{{\rm{SM}}}_{D}^{E}}{\sigma }_{{{\rm{SM}}}_{D}^{O}}},\left\{\begin{array}{l}{\rm{cov\; :\; covariance}}\\ \sigma {\rm{:\; standard}}\;{\rm{deviation}}\end{array}\right.$$

14 $${\rm{GCF}}=\frac{{\rm{Area}}\;{\rm{of}}\;{\rm{successful}}\;{{\rm{SM}}}_{{\rm{t}}}^{{\rm{E}}}\;{\rm{retrievals}}}{{\rm{The}}\;{\rm{total}}\;{\rm{area}}\;{\rm{of}}\;{\rm{land}}\;{\rm{surface}}}$$

where ${{\rm{SM}}}_{D}^{E}$ (cm^3^/cm^3^) is the estimated soil moisture value on day *D*; ${{\rm{SM}}}_{D}^{O}$ (cm^3^/cm^3^) is the *in-situ* measured soil moisture on day *D*; *N* is the number of measurements.

## Data Records

Global daily-scale soil moisture fusion dataset (GDSMFD) based on TCA-LWF (2011–2018) is publicly available at the National Tibetan Plateau Data Center (10.11888/Terre.tpdc.271935)^54^.This dataset contains global daily-scale soil moisture fusion data with a spatial grid resolution of 25 km, in cm^3^/cm^3^, from January 1, 2011 to December 31, 2018. These data are stored in TIFF format with one file per day. Each file is named as “Fusion_SMOS_FY3B_ASCAT_ESACCI_SMAP_V1_yymmdd.tif”, where “yy” represents the year, “mm” represents the month and “dd” represents the day.

## Technical Validation

### Verification of TCA assumptions

The SMOS, FY3-B, and ASCAT were used to generate the 1^st^ merged data product in the first step (Table 3), then combined the latter with SMAP and ESA-CCI in the second step (Table 3). TCA assumes independent errors of each pair of datasets. So, in each triplet, we selected soil moisture products generated with algorithms as different as possible, because algorithm similarity may lead to correlated errors on the SSM datasets (Table 3). In triplet 1, SMOS SSM product was retrieved using the L-MEB algorithm from the MIRAS passive microwave brightness temperature observations with L band (1.4 GHz) and multi-angles (0~55°), FY3-B SSM product was estimated using the Qp- algorithm from the MWRI passive microwave brightness temperature observations with X band (10.65 GHz) and single incidence angle (55°), while the ASCAT SSM product was retrieved using the SWI algorithm from active microwave backscattering observations in C band (5.255 GHz) and multi-angular observations (25~65°). In triplet 2, the 1^st^ merged SSM product was estimated using TCA-based LWF algorithm from SMOS, FY3-B and ASCAT SSM products, while SMAP SSM product was retrieved using the SCA-V algorithm from L band (1.4GHz) and single incidence angle (40°) and the ESA-CCI SSM product was derived from seven passive microwave SSM products (SMMR, SSM/I, TRMM, AMSR-E, AMSR-2, and SMOS) using the LPRM algorithm, and three active microwave SSM products (ERS-1/2, MetOp-A/ASCAT, and MetOp-B/ASCAT) using the change detection algorithm.

In Eq. 2 to Eq. 3, Cov(*ε*_*i*_, *ε*_*j*_) = 0 when *i* ≠ *j* (zero error cross-correlation) and Cov(*t*, *ε*_*i*_) = 0 (error-orthogonality) were assumed. To verify quantitatively the assumptions of TCA when the pairing strategy of SMOS, FY3-B, ASCAT, SMAP and ESA-CCI (i.e., Table 3) was used to apply the TCA algorithm, the *in-situ* soil moisture measurement data of CTP_SMTMN, RSMN, DAHRA, BIEBRZA_S-1, MySMNet and REMEDHUS networks were considered the “true” SSM values, i.e., the *t* in Eqs. 1~3). To calculate the errors on the SSM products, i.e., the *ε* in Eq. 1, first, the linear function *S*SM = *α* + β*t between the *in-situ* soil moisture measurement and soil moisture product was fitted and the values of the *α* and *β* parameters were obtained. Second, the rescaled “true” values (SSM′) were calculated using *in-situ* soil moisture measurements based on the fitted linear equation. Finally, according to Eq. 1, the *ε* values of each soil moisture product were expressed as *ε* = *S*SM−SSM′ (Table 5) i.e., the difference between the original satellite SSM soil moisture product value and the rescaled “true” values.

**Table 5:** Covariance (cm^6^/cm^6^) and correlation values between errors of each pair of soil moisture products in triplet 1 and 2 (i.e., the Cov(*ε*_*i*_, *ε*_*j*_) and *R*^2^ values of Eq. 2), and covariance values between *in-situ* soil moisture measurements and errors of soil moisture products (i.e., the Cov(*t*, *ε*_*i*_) and Cov(*t*, *ε*_*j*_) values of Eq. 2).

	Pairs (*i*, *j*)	Cov(*t*, *ε*_*i*_)	Cov(*t*, *ε*_*j*_)	Cov(*ε*_*i*_, *ε*_*j*_)	*R*^2^
Triplet 1	SMOS, FY3-B	2.87*10^−04^	−2.48*10^−05^	4.88*10^−04^	0.005
	SMOS, ASCAT	2.87*10^−04^	−2.77*10^−07^	2.66*10^−03^	0.139
	FY3-B, ASCAT	−2.48*10^−05^	−2.77*10^−07^	1.74*10^−03^	0.081
Triplet 2	1st merged SSM, ESA-CCI	−7.46*10^−08^	8.37*10^−08^	8.46*10^−04^	0.014
	1st merged SSM, SMAP	−7.46*10^−08^	1.71*10^−07^	2.15*10^−03^	0.054
	ESA-CCI, SMAP	8.37*10^−08^	1.71*10^−07^	3.52*10^−03^	0.215

From Table 5, all absolute Cov(*t*, *ε*_*i*_) and Cov(*t*, *ε*_*j*_) values of triplet 1 and 2 are less than 0.00029 cm^6^/cm^6^, and most of Cov(*t*, *ε*_*i*_) and Cov(*t*, *ε*_*j*_) only are approximately 0. All Cov(*ε*_*i*_, *ε*_*j*_) values of triplet 1 and 2 are less than 0.00352 cm^6^/cm^6^. The minimum value of Cov(*ε*_*i*_, *ε*_*j*_) equal to 0.00049 cm^6^/cm^6^, occurred in the pair ESA-CCI and SMAP SSM. Comparing the absolute Cov(*t*, *ε*_*i*_) (or Cov(*t*, *ε*_*j*_)) and Cov(*ε*_*i*_, *ε*_*j*_) values, the absolute Cov(*t*, *ε*_*i*_) (or Cov(*t*, *ε*_*j*_)) are significantly lower than the Cov(*ε*_*i*_, *ε*_*j*_) values i.e., the influence of error non-orthogonality is smaller than the error cross-correlation. In addition, the *R*^2^ values between errors of each pair of soil moisture products in triplet 1 and 2 are lower. Only the *R*^2^ values between ESA-CCI and SMAP, SMOS and ASCAT are higher, 0.215 and 0.139 respectively. Therefore, in this study the estimated Cov(*ε*_*i*_, *ε*_*j*_), Cov(*t*, *ε*_*i*_) and Cov(*t*, *ε*_*j*_) values using *in-situ* measurements when the pair strategy of SMOS, FY3-B, ASCAT, SMAP and ESA-CCI (i.e., Table 3) was used to apply the TCA algorithm were negligible suggesting the TCA assumptions did apply to our data.

### Global-scale weight comparison

**Fig. 4:**
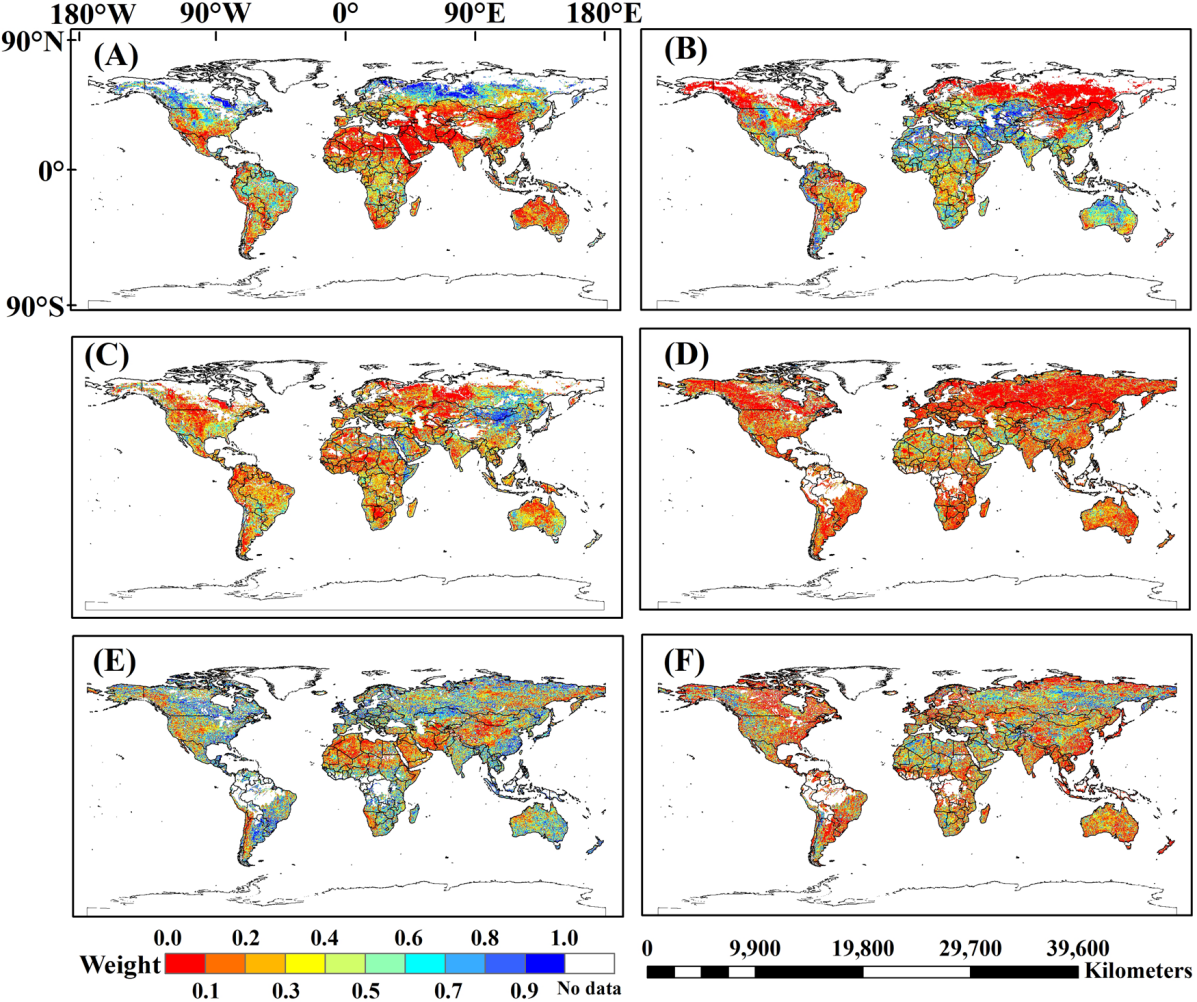
Weight values of SMOS (**A**), FY3-B (**B**), ASCAT (**C**), the 1^st^ merged SSM (**D**), ESA-CCI (**E**), and SMAP (**F**) SSM products based on TCA.

First, we calculated the weight values of SMOS, FY3-B and ASCAT SSM products based on TCA (Eqs. 1~8), and merged these three SSM products by applying the linear weight fusion method with the weight values to get the 1^st^ merged SSM product (Table 4). Then, we used the first merged SSM product, ESA-CCI, and SMAP SSM in the same way. The global-scale weight distribution maps of SMOS, FY3-B, ASCAT, ESA-CCI, and SMAP SSM products are compared and shown in Fig. 4.

In different areas, the weight values of SMOS, FY3-B, and ASCAT have a large spatial variability (Fig. 4). There are some areas without data because of less sample points used in TCA calculation in triplet 1 or 2 and permanent snow, ice and water bodies. In Australia and northern Africa, the weight value of FY3-B is higher than that of SMOS and ASCAT, which means that FY3-B is a dominant SSM product in Australia and North Africa when merging SMOS, FY3-B, and ASCAT SSM products. The RMSE of FY3-B SSM calculated by TCA (Eq. 6) is lower than that of SMOS and ASCAT. In northern Asia, northern America and eastern South America, the weight values of SMOS are higher than that of FY3-B and ASCAT and SMOS is a dominant SSM product in the merging process. In general, SMOS and FY3-B are dominant SSM products in the first merging process. Comparing weight values of the 1^st^ merged SSM product, ESA-CCI, and SMAP SSM, the weight values of ESA-CCI and SMAP are higher than that of the 1^st^ merged SSM product. Especially, in Australia, Asia, and most of America, the weight values of ESA-CCI are higher but lower than SMAP in most of northern Africa. The weight values of 1^st^ merged SSM product are lower in most of areas (Fig. 4D). Lower weight value does represent that the RMSE values of 1^st^ merged SSM product estimated based on TCA are higher than RMSE values of ESA-CCI and SMAP SSM products. There maybe are more potential errors in 1^st^ merged SSM data. In 2^nd^ merged SSM data, the large errors of 1^st^ merged SSM data may be reduced by using lower weight values of 1^st^ merged SSM data.

### Global-scale comparison of SSM products

**Fig. 5:**
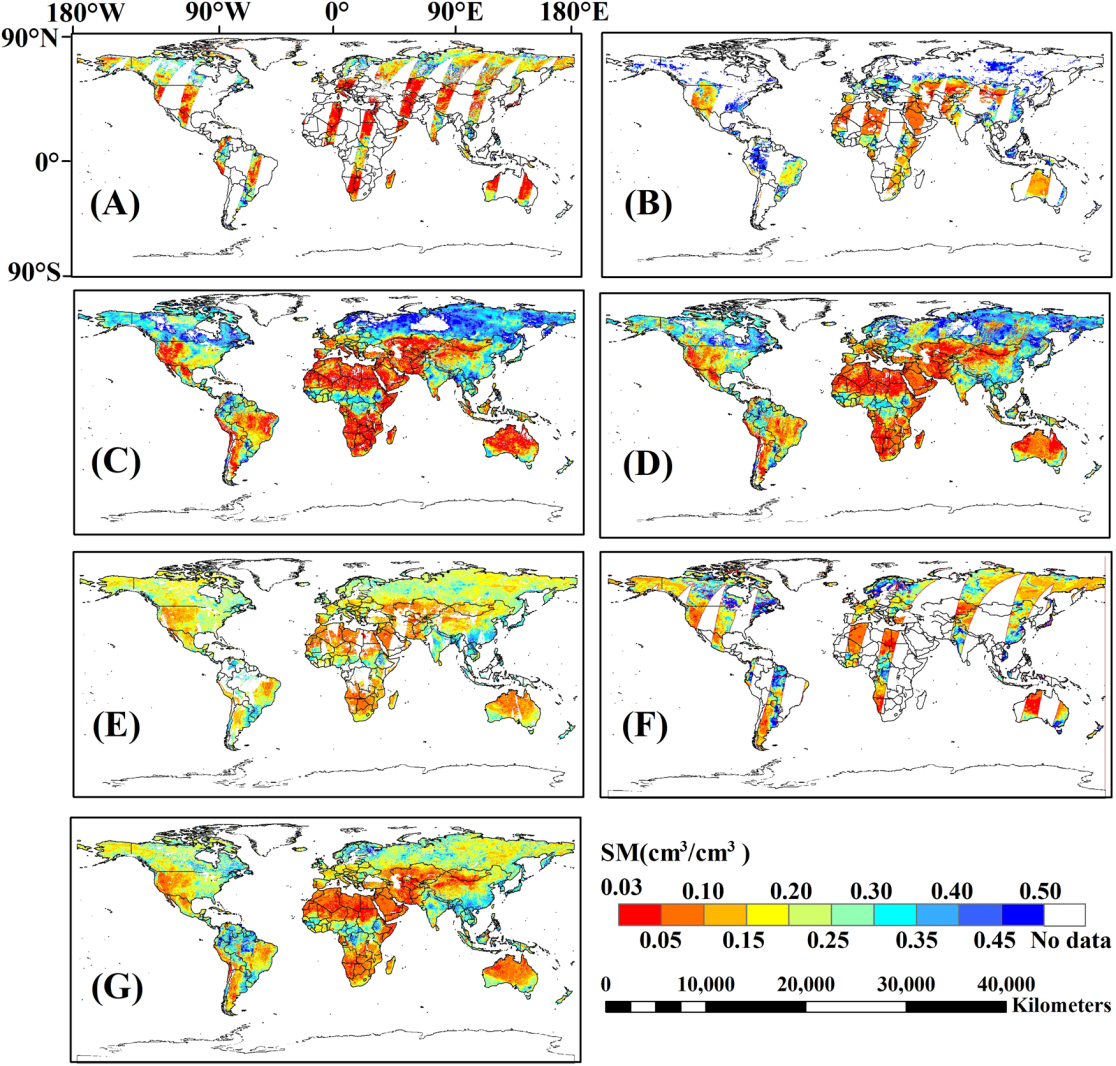
Global SSM data products on August 1^st^, 2015: SMOS (**A**), FY3-B (**B**), ASCAT (**C**), 1^st^ merged SSM (**D**), ESA-CCI (**E**), SMAP (**F**) and 2^nd^ merged SSM (**G**).

**Fig. 6:**
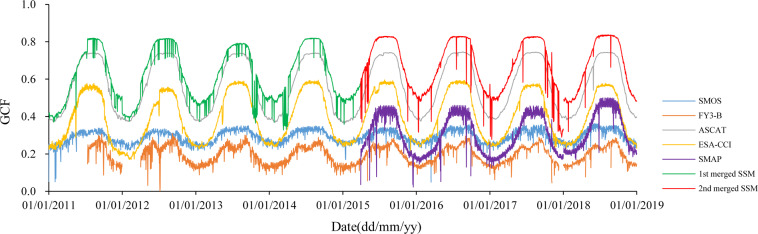
Daily GCF during 2011~2018 of global SMOS, FY3-B, ASCAT, ESA-CCI, SMAP, 1^st^ and 2^nd^ merged SSM (2015~2018 only).

We compared the spatial distribution of global SMOS, FY3-B, ASCAT, ESA-CCI, SMAP, the 1^st^ and 2^nd^ merged SSM products on August 1, 2015 (Fig. 5). The areas of permanent snow, ice and water bodies were filtered and masked in the global SSM products using the ESA-CCI L4 2015 global LC map with 300 m SGR. Besides, we quantitatively compared the GCF values (Eq. 14) of SMOS, FY3-B, ASCAT, ESA-CCI, SMAP, the 1^st^ and 2^nd^ merged SSM products from 2011 to 2018 (Table 6 and Fig. 6).

**Table 6:** Min, max and mean GCF of global SMOS, FY3-B, ASCAT, ESA-CCI, SMAP, 1^st^ and 2^nd^ merged SSM products from 2011 to 2018.

GCF	SMOS	FY3-B	ASCAT	1^st^ merged SSM	ESA-CCI	SMAP	2^nd^ merged SSM
Min	0.049	0.002	0.328	0.360	0.170	0.022	0.278
Max	0.363	0.310	0.745	0.820	0.596	0.501	0.837
Average	0.291	0.195	0.567	0.616	0.398	0.308	0.672

Missing values in the 2^nd^ merged SSM product are significantly less than in SMOS, FY3-B, ASCAT, ESA-CCI, and SMAP SSM (Fig. 5). The 2^nd^ merged SSM product has almost complete coverage around the globe. In northern Asia and America, the values of the 2^nd^ merged SSM product are lower than the 1^st^ merged SSM. In northern Africa the 2^nd^ merged SSM is lower than the ESA-CCI SSM. In northern Asia, most values of SMOS, ESA-CCI, SMAP, and 2^nd^ merged SSM are in the range of 0.05~0.35 cm^3^/cm^3^, while most values of the ASCAT and 1^st^ merged SSM are >0.35 cm^3^/cm^3^. There are many missing values in the FY3-B SSM in northern Asia (Fig. 5B). In northern Africa, the values of the 2^nd^ merged SSM and SMAP SSM data products are between 0.03 and 0.15 cm^3^/cm^3^, while the ESA-CCI SSM is in the range of 0.05~0.20 cm^3^/cm^3^.

The GCF of the 1^st^ and the 2^nd^ merged SSM is higher than that of SMOS, FY3-B, ASCAT, ESA-CCI, and SMAP (Fig. 6), since gaps in a component data set are filled using retrievals in the remaining components of a triplet. The mean and maximum GCF of the 2^nd^ merged SSM product are the highest ones, i.e., 0.67 and 0.84 respectively (Table 6). Clearly, the GCF of all SSM data products is highest in the rainy season from June to September and lowest in the dry season in January, especially for ASCAT, ESA-CCI, SMAP, 1^st^ and 2^nd^ merged SSM. The intra-annual GCF amplitude of SMOS and FY3-B SSM is smaller than that of ASCAT, ESA-CCI, SMAP, 1^st^ and 2^nd^ merged SSM. The GCF of SMOS SSM fluctuates up and down around 0.25. Comparing with GCF values of SMOS, ASCAT and SMAP retrieved from observations of single satellite sensor, the GCF of FY3-B were the lowest, and the max GCF of FY3-B was only 0.31. Overall, in terms of global coverage, the 1^st^ and 2^nd^ merged SSM products were improved using the TCA- LWF algorithm, particularly the 2^nd^ merged SSM product.

### Comparison with *in-situ* measurements

**Fig. 7:**
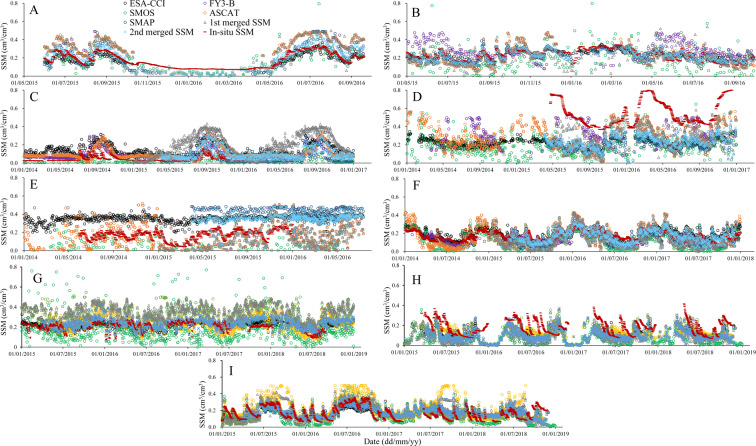
Temporal evolution of *in-situ*, SMOS, FY3-B, ASCAT, ESA-CCI, SMAP, the 1^st^ and 2^nd^ merged SSM in the seven reference areas ((**A**) CTP_SMTMN; (**B**) RSMN; (**C**) AMMA-CATCH and DAHRA; (**D**) BIEBRZA_S-1; (**E**) MySMNet; (**F**) REMEDHUS; (**G**) HOBE; (**H**) USCRN; (**I**) OZNET).

**Fig. 8:**
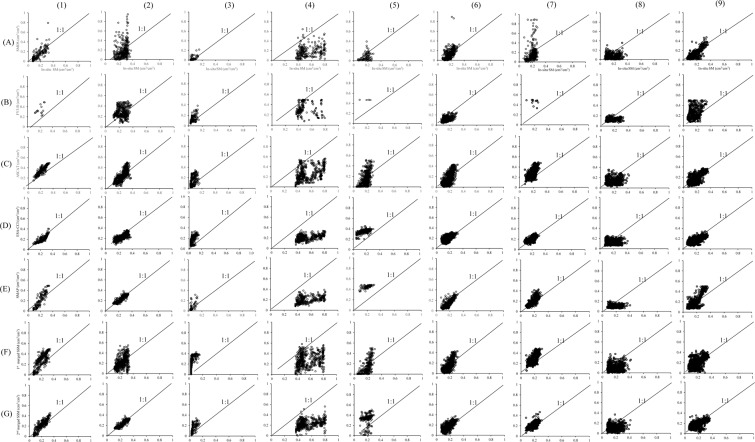
Scatter plots of SSM retrievals: SMOS (**A**), FY3-B (**B**), ASCAT (**C**), ESA-CCI (**D**), SMAP (**E**), 1^st^ merged (**F**) and 2^nd^ merged (**G**) versus *in-situ* soil moisture measurements vs at: (1) CTP_SMTMN, (2) RSMN, (3) AMMA-CATCH and DAHRA, (4) BIEBRZA_S-1, (5) MySMNet, (6) REMEDHUS, (7) HOBE, (8) USCRN, (9) OZNET.

We used the mean value of *in-situ* measurements within a satellite SSM pixel as the reference to evaluate the satellite SSM products. We compared the SMOS, FY3-B, ASCAT, ESA-CCI, SMAP, the 1^st^ and 2^nd^ merged SSM with *in-situ* soil moisture measurements from 2014 to 2018 (Figs. 7, 8). In CTP_SMTMN (China), AMMA-CATCH (Benin, Niger, Mali, and DAHRA (Senegal) the soil moisture measurements from 2015 to 2016 show a clear seasonality with higher SM in the rainy season and lower in the dry season. The seasonality was also very clear in BIEBRZA_S-1 (Poland), HOBE (Denmark) and REMEDHUS (Spain), where the soil moisture was higher in winter and lower in summer especially in BIEBRZA_S-1. In RSMN (Romania) and MySMNet (Malaysia) there was no clear seasonality from 2014 to 2017. In RSMN and HOBE the soil moisture from 2014 to 2018 fluctuated around 0.2 cm^3^/cm^3^ and 0.25 cm^3^/cm^3^ respectively. In USCRN (USA) and OZNET (Australia), there were many situations when the soil moisture increased sharply from 2015 to 2018, possibly in response irrigation. Overall, the 2nd merged SSM product could capture the soil moisture dynamic characteristics caused by rainfall or irrigation, especially in CTP_SMTMN, REMEDHUS and OZNET, and was basically consistent with the temporal variability of *in-situ* soil moisture.

In CTP_SMTMN and RSMN, the 2^nd^ merged SSM product was more consistent with *in-situ* soil moisture measurements especially from April to September. In winter from October to March of next year, however, the 2^nd^ merged SSM underestimated the soil moisture compared with the *in-situ* measurements. The 1^st^ merged SSM underestimated soil moisture in winter and largely overestimated in summer. In these areas the ESA-CCI SSM product performed better than SMOS, FY3-B, ASCAT, SMAP and the 1^st^ merged SSM. In AMMA-CATCH and DAHRA most SSM retrievals from 2014 to 2017 were largely overestimated, especially in the rainy season, except SMOS and SMAP SSM. In these areas, most SMOS SSM retrievals underestimated soil moisture. The SMAP SSM was more consistent with *in-situ* soil moisture measurements, although part of the SMAP retrievals were overestimated. In the BIEBRZA_S-1 area, almost all SSM retrievals from 2015 to 2017 were underestimated. In the BIEBRZA_S-1 area from 2015 to 2017, the mean *in-situ* soil moisture was larger than 0.4 cm^3^/cm^3^ and the highest soil moisture measurement was as high as about 0.8 cm^3^/cm^3^. The *in-situ* soil moisture measurements, therefore, may not be reliable here. In the MySMNet area, most SMOS SSM retrievals were underestimated. There were many missing soil moisture retrievals in FY3-B SSM data product in the MySMNet area. The reason may be the limited FY3-B MWRI observation range and the soil moisture retrieval algorithm applied to the FY3-B radiometric data. The ESA-CCI and SMAP SSM largely overestimated the soil moisture compared with the *in-situ* measurements.

Overall, in the MySMNet area from 2014 to 2016 all SSM retrievals performed badly against *in-situ* soil moisture measurements, while performed better in the REMEDHUS area. ESA-CCI, SMAP and the 2^nd^ merged SSM were more consistent with *in-situ* soil moisture measurements. The SMOS SSM product largely underestimated soil moisture from 2014 to 2018, especially in the rainy season. In the same period the ASCAT SSM underestimated in the rainy season and overestimated soil moisture in the dry season. Overall, in the CTP_SMTMN, RSMN, and REMEDHUS areas, the ESA-CCI, SMAP and 2^nd^ merged SSM was more consistent with *in-situ* soil moisture measurements.

We applied the MAE (Eq. 10), RMSE (Eq. 11), RB (Eq. 12) and *R* (Eq. 13) metrics to evaluate the SMOS, FY3-B, ASCAT, ESA-CCI, SMAP, the 1^st^ and 2^nd^ merged SSM against the *in-situ* soil moisture measurements at the selected sites from the ISMN (Table 7).

**Table 7:** MAE (cm^3^/cm^3^), RMSE (cm^3^/cm^3^), RB and *R* values of SMOS, FY3-B, ASCAT, ESA-CCI, SMAP, the 1st and 2nd merged SSM against *in-situ* soil moisture measurements from ISMN. (ACD: AMMA-CATCH and DAHRA networks).

Indies	Networks	SMOS	FY3-B	ASCAT	1^st^ merged SSM	ESA-CCI	SMAP	2^nd^ merged SSM
MAE	CTP_SMTMN	0.102	0.120	0.102	0.098	0.070	0.074	0.064
	RSMN	0.107	0.100	0.109	0.075	0.032	0.028	0.028
	ACD	0.033	0.053	0.063	0.059	0.088	0.024	0.048
	BIEBRZA_S-1	0.381	0.156	0.298	0.276	0.338	0.303	0.287
	MySMNet	0.091	/	0.087	0.094	0.181	0.207	0.152
	REMEDHUS	0.075	0.065	0.071	0.071	0.038	0.049	0.040
	HOBE	0.134	0.282	0.135	0.141	0.060	0.071	0.072
	USCRN	0.094	0.138	0.095	0.095	0.079	0.099	0.085
	OZNET	0.064	0.138	0.067	0.080	0.061	0.086	0.072
	***Mean***	***0.120***	***0.132***	***0.114***	***0.110***	***0.105***	***0.105***	***0.094***
RMSE	CTP_SMTMN	0.124	0.140	0.115	0.114	0.080	0.090	0.075
	RSMN	0.126	0.120	0.150	0.098	0.041	0.036	0.036
	ACD	0.038	0.065	0.086	0.075	0.104	0.043	0.069
	BIEBRZA_S-1	0.410	0.221	0.340	0.315	0.360	0.331	0.313
	MySMNet	0.109	/	0.108	0.109	0.186	0.216	0.161
	REMEDHUS	0.093	0.082	0.082	0.083	0.049	0.058	0.051
	HOBE	0.188	0.291	0.155	0.161	0.074	0.085	0.087
	USCRN	0.112	0.158	0.112	0.114	0.092	0.111	0.101
	OZNET	0.082	0.175	0.083	0.099	0.076	0.109	0.088
	***Mean***	***0.142***	***0.157***	***0.137***	***0.130***	***0.118***	***0.120***	***0.109***
RB	CTP_SMTMN	0.382	0.290	0.258	0.258	0.180	0.058	0.053
	RSMN	0.437	0.200	0.400	0.048	0.065	0.019	0.068
	ACD	0.083	0.553	0.573	0.561	0.671	0.185	0.493
	BIEBRZA_S-1	−1.895	−0.379	−1.122	−0.907	−1.596	−1.280	−1.372
	MySMNet	−0.468	/	0.075	0.272	0.516	0.522	0.382
	REMEDHUS	−0.449	−0.449	−0.187	−0.257	0.125	−0.283	−0.013
	HOBE	−0.119	0.572	0.315	0.330	0.010	0.056	0.126
	USCRN	−0.274	0.273	−0.076	0.001	0.112	0.053	0.081
	OZNET	−0.039	0.459	0.189	0.229	0.133	0.255	0.141
	***Mean***	***−0.260***	***0.190***	***0.047***	***0.059***	***0.024***	***−0.046***	***−0.005***
*R*	CTP_SMTMN	0.639	0.560	0.806	0.819	0.790	0.824	0.766
	RSMN	0.497	0.110	0.266	0.493	0.772	0.821	0.861
	ACD	0.644	0.770	0.595	0.590	0.699	0.750	0.718
	BIEBRZA_S-1	0.293	−0.028	0.371	0.319	0.445	0.528	0.331
	MySMNet	0.385	/	0.646	0.635	0.599	0.450	0.398
	REMEDHUS	0.637	0.372	0.744	0.729	0.708	0.803	0.691
	HOBE	0.155	−0.162	0.540	0.499	0.541	0.696	0.633
	USCRN	0.522	0.015	0.455	0.366	0.528	0.627	0.573
	OZNET	0.743	0.443	0.736	0.626	0.720	0.775	0.730
	***Mean***	***0.502***	***0.260***	***0.573***	***0.564***	***0.645***	***0.697***	***0.633***

The 2^nd^ merged and the SMAP SSM were more consistent with the CTP_SMTMN and RSMN soil moisture measurements. The lowest MAE, RMSE, RB values, and highest *R*-value were 0.028 cm^3^/cm^3^, 0.036 cm^3^/cm^3^, 0.019, and 0.861 respectively. In the AMMA-CATCH and DAHRA networks of Africa, the FY3-B, SMAP, and SMOS SSM retrievals were more consistent with *in-situ* soil moisture measurements than ESA-CCI, 1^st^ and 2^nd^ merged SSM. The MAE value was lowest for SMAP, i.e., 0.024 cm^3^/cm^3^. The RMSE and RB values of SMOS were the lowest, i.e., 0.038 cm^3^/cm^3^ and 0.083 respectively. The *R*-value was highest for FY3-B/MWRI, i.e., 0.770. The 2^nd^ merged SSM was more consistent than ESA-CCI with the AMMA-CATCH and DAHRA soil moisture measurements. In BIEBRZA_S-1 and MySMNet networks, the difference between soil moisture measurements and all SSM retrievals was very large. Especially, in the BIEBRZA_S-1 network, all SSM products performed very badly, largely underestimating measured soil moisture. In the REMEDHUS network, the ESA-CCI, SMAP, and 2^nd^ merged SSM were more consistent with soil moisture measurements. The MAE and RMSE were lowest for ESA-CCI, i.e., 0.038 cm^3^/cm^3^ and 0.049 cm^3^/cm^3^ respectively. The RB value was lowest for the 2^nd^ merged SSM, i.e., −0.013, while the *R*-value was highest for SMAP, i.e., 0.803. In HOBE, USCRN and OZNET networks, although the MAE and RMSE values of 2^nd^ merged SSM were slightly larger than ESA-CCI SSM, the MAE and RMSE values of 2^nd^ merged SSM were clearly less than SMOS, FY3-B, ASCAT, 1^st^ merged and SMAP SSM.

Overall, in the 10 networks CTP_SMTMN, RSMN, AMMA-CATCH, DAHRA, BIEBRZA_S-1, MySMNet, REMEDHUS, HOBE, USCRN and OZNET, the mean values of MAE, RMSE and BR values of the 2^nd^ merged SSM were lower than SMOS, FY3-B, ASCAT, ESA-CCI, SMAP and 1^st^ merged SSM (Table 7). For example, the mean of all MAE values of 2^nd^ merged SSM was 0.094 cm^3^/cm^3^ less than 0.120 cm^3^/cm^3^ of SMOS, 0.132 cm^3^/cm^3^ of FY3-B, 0.114 cm^3^/cm^3^ of ASCAT, 0.110 cm^3^/cm^3^ of 1^st^ merged SSM, 0.105 cm^3^/cm^3^ of ESA-CCI and 0.105 cm^3^/cm^3^ of SMAP. Although, the mean value (0.633) of all *R* values of 2^nd^ merged SSM was less than ESA-CCI (0.645) and SMAP (0.697), it was higher than SMOS (0.502), FY3-B (0.260), ASCAT (0.573) and 1^st^ merged SSM (0.564). In addition, except the MySMNet and BIEBRZA_S-1 networks, the 2^nd^ merged SSM was more consistent with soil moisture measurements than the 1^st^ merged SSM. It should be noted that in AMMA-CATCH and DAHRA networks, the SMOS and SMAP SSM retrievals from observations by a single microwave radiometer were more consistent with *in-situ* soil moisture measurements than the ESA-CCI, 1^st^ and 2^nd^ merged SSM. Overall, the 2^nd^ merged SSM was more consistent than ESA-CCI with *in-situ* soil moisture measurements at most sites.

To improve our validation study on GDSMFD product, we have expanded significantly the soil moisture reference data set used for this purpose (see the Supplementary file). We used soil moisture retrievals based on the area-scale airborne radiometer observation by SCA-V (Single Channel Algorithm-Vertical polarization) soil moisture retrieval algorithm. Also, we used two global-scale merged soil moisture data, i.e., the NNsm and RSSSM data products based on the neural network fusion algorithm. We deemed potentially confusing to mix the validation against actual soil moisture measurements with the comparison with retrievals and other data products. Accordingly, the comparison of our merged soil moisture data of this study with the airborne retrievals and the NNsm and RSSSM data products are presented in the document on supplementary materials. Comparing with the airborne radiometer observation-based soil moisture data, the 2^nd^ merged soil moisture product had better performance in term of consistence with the airborne radiometer observation-based soil moisture data than FY3-B, ASCAT, ESA-CCI and 1^st^ merged soil moisture products. In addition, the spatial distribution pattern of GDSMFD product has basically consistent spatial pattern with vegetation coverage data in global scale, and also is same with NNsm and RSSSM products.

## Usage Notes

**Fig. 9:**
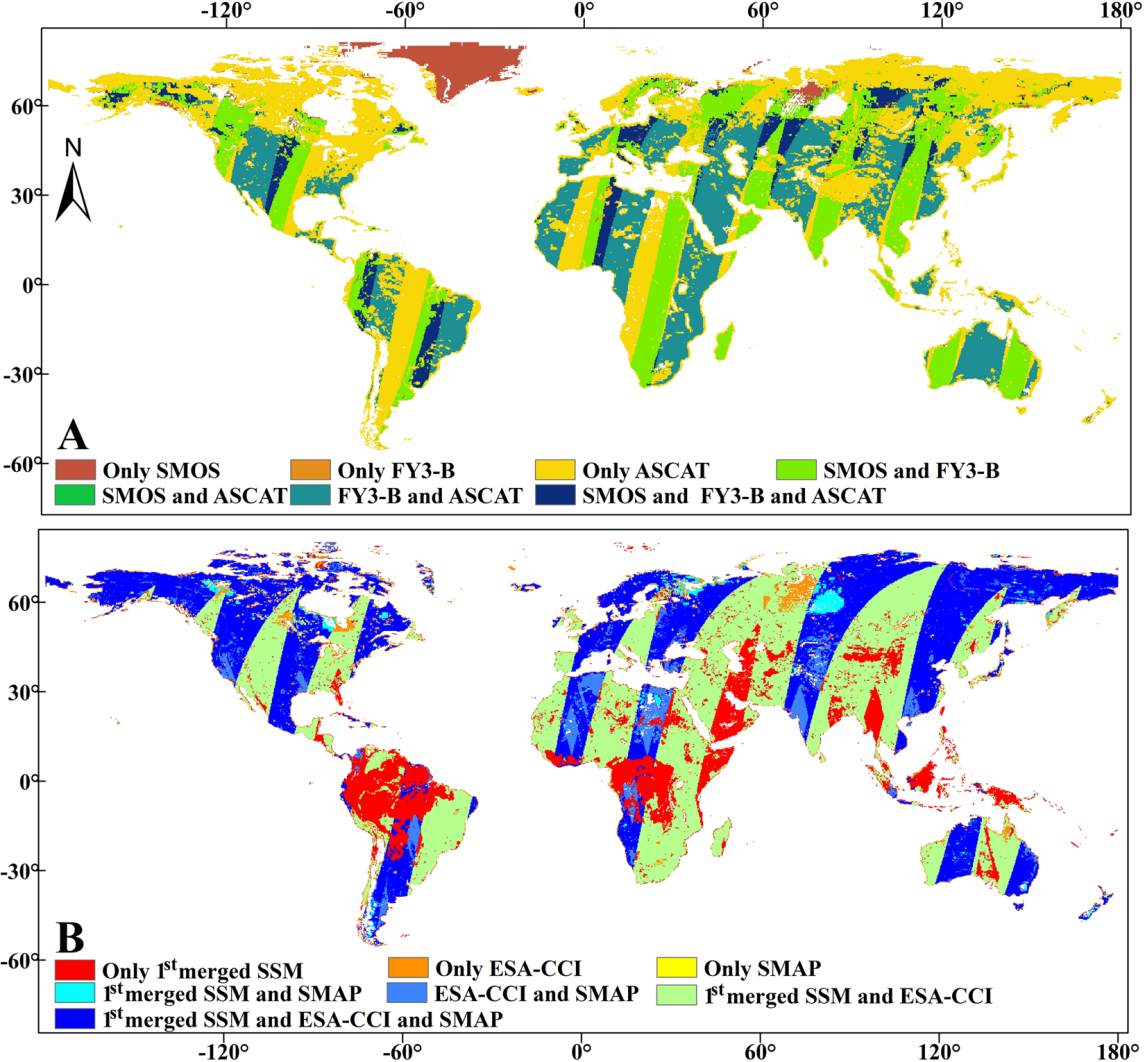
Distribution flags of SMOS, FY3-B, ASCAT, ESA-CCI and SMAP SSM data on August 1, 2015 in: (**A**) the 1^st^ merged SSM data; (**B**) the 2^nd^ merged SSM data.

We present a global daily-scale soil moisture fusion dataset generated through the TCA-LWF. It is possible that in any given pixel only one or two of SMOS, FY3-B and ASCAT SSM is available for 1^st^ merged SSM data, or only one or two of 1^st^ merged SSM, ESA-CCI and SMAP SSM data is available for the 2^nd^ merged SSM data. Therefore, it is helpful to note the spatial distribution of SSM data products for use and further improvement of the merged SSM generated by this study in future. Taking August 1^st^, 2015 as an example, we produced the distribution flags of SMOS, FY3-B and ASCAT SSM data for the 1^st^ merged SSM product (Fig. 9A), and the distribution flags of the 1^st^ merged SSM, ESA-CCI and SMAP SSM data for the 2^nd^ merged SSM product (Fig. 9B). The areas where SMOS, FY3-B and ASCAT SSM data overlap are smaller than the areas where either SMOS and FY3-B or FY3-B and ASCAT SSM data do (Fig. 9A). In addition, there are many areas where only ASCAT SSM is available (Yellow areas in Fig. 9A). In these areas, the errors in the 1^st^ merged SSM data are only due to errors in the ASCAT SSM data. In the 2^nd^ merged SSM data, there are many areas with overlapping coverage (Deep blue areas in Fig. 9B) by the 1^st^ merged SSM, ESA-CCI and SMAP and by the 1^st^ merged SSM and ESA-CCI (Green areas in Fig. 9B). In northern South America and central Africa (Red areas in Fig. 9B), there is only the 1^st^ merged SSM data available i.e., in these areas the errors of 2^nd^ merged SSM data only come from the 1^st^ merged SSM data.

In addition, although the final 2^nd^ merged SSM product has better performance in accuracy than SMOS, FY3-B, ASCAT, SMAP and even than ESA-CCI SSM (Fig. 6 and Table 7), there are overestimations or underestimations against *in-situ* soil moisture measurements especially with the data collected in the AMMA-CATCH and DAHRA, BIEBRZA_S-1 and MySMNet networks. According to the LWF algorithm (Eqs. 7~9), the errors leading to overestimations or underestimations in the final 2^nd^ merged SSM are from SMOS, FY3-B, ASCAT, ESA-CCI and SMAP SSM. Therefore, for further improvement of the merged SSM product generated by this study, it is also necessary to discuss the potential error sources in SSM data products, which is dealt with in the following sections.

### Errors derived from ESA-CCI SSM

We found that the SMOS, FY3-B and SMAP SSM retrievals from observations by a single microwave radiometer were more consistent with *in-situ* soil moisture measurements in AMMA-CATCH and DAHRA than the ESA-CCI SSM (Figs. 7 and 8). Most soil moisture values of ESA-CCI SSM are overestimated compared with *in-situ* soil moisture measurements in AMMA-CATCH and DAHRA especially in the rainy season. The calculated RMSE value (0.104 cm^3^/cm^3^) of ESA-CCI SSM (Table 7) is higher than the RMSE values of SMOS, FY3-B and SMAP, i.e., 0.038, 0.065 and 0.043 cm^3^/cm^3^ respectively. In addition, most ESA-CCI SSM weight values were lower, less than 0.3 or even less than 0.1 in northern Africa, than the SMAP SSM weight values, which were higher than 0.3 (Fig. 4). The weight values were calculated by Eqs. 7 and 8 with the RMSE values estimated using TCA and decrease with increasing RMSE. In the ESA-CCI SSM data product, all the SSM retrievals with passive microwave data such as AMSR-E, SMOS and AMSR-2 SSM were done with the same retrieval algorithm i.e., LPRM. Some authors indicated however, that the AMSR-E, AMSR-2 and SMOS SSM retrievals with LRPM overestimated the soil moisture, and were inconsistent with *in-situ* soil moisture measurements^9,26,55–57^. For example, Cui *et al*.^26^ found that the AMSR-2 SSM retrieved using LPRM largely overestimated the soil moisture with bias >0.09 cm^3^/cm^3^ in the Little Washita Watershed and REMEDHUS networks. It was also indicated that the vegetation optical depth was overestimated by LPRM leading to overestimation of soil moisture retrieved using LPRM^26^. In AMMA-CATCH and DAHRA network, the main land cover type is shrubland with sparse vegetation and low vegetation optical depth. Accordingly, one of the reasons of overestimation of ESA-CCI SSM data in northern Africa is the overestimation of vegetation optical depth by LPRM. Therefore, in areas with low vegetation, the LPRM soil moisture retrieval may not be the most accurate. The LPRM may lead to errors introduced into the ESA-CCI product, because LPRM does not perform well in global SSM retrieval with microwave radiometers. So, it is not unlikely that errors caused by LPRM in the ESA-CCI SSM data product were introduced in our 2^nd^ merged SSM data.

### Errors derived from ASCAT SSM

**Fig. 10:**
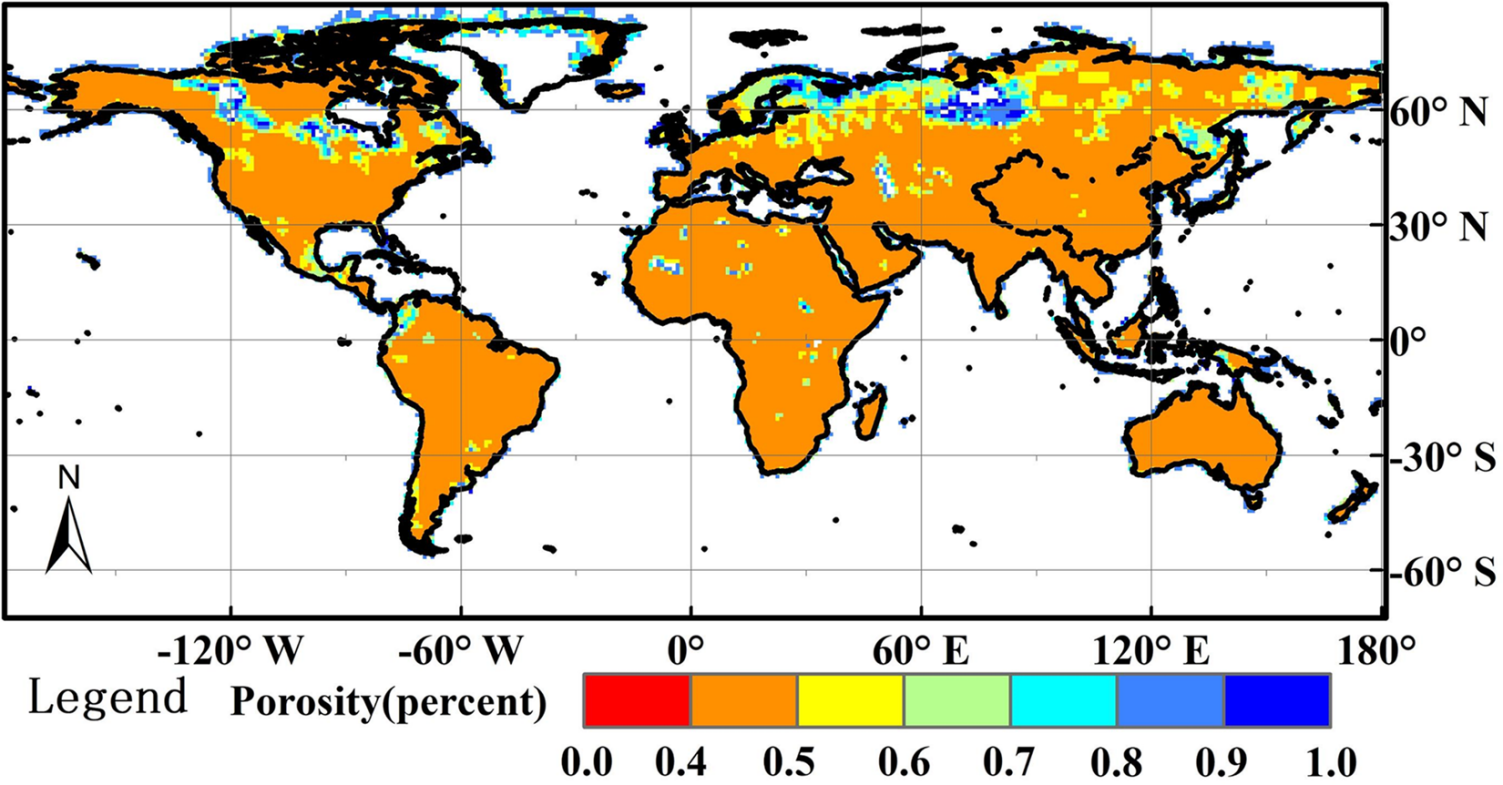
Global soil porosity map^53^.

The GCF of ASCAT SSM is higher than SMOS, FY3-B, SMAP and even than ESA-CCI (Fig. 6). There are many areas where only ASCAT SSM is available (Fig. 9). Therefore, ASCAT SSM plays an important role in our 1^st^ and 2^nd^ merged SSM data, especially in the 1^st^ merged SSM. From ASCAT SSM product retrieved using SWI algorithm with C-band observations (5.3 GHz) gave a worse performance (Table 7) than SMOS, FY3-B and SMAP SSM and overestimated soil moisture compared with the *in-situ* measurements in the CTP_SMTMN, RSMN, AMMA-CATCH and DAHRA, MySMNet networks. Other studies drew similar conclusions, i.e. although ASCAT SSM was correlated with *in-situ* soil moisture data, there was an issue of overestimation against *in-situ* soil moisture measurements^9^. One possible cause leading to overestimation in the ASCAT SSM could be errors in the global soil porosity data with 1-degree SGR used to convert the ASCAT SSM from degree of saturation (%) into volumetric soil moisture content (cm^3^/cm^3^)^10,58,59^. In this study, the adopted soil porosity data (Fig. 10) was estimated using equations developed by Saxton and Rawls (2006), who took sand, clay, silt, and organic matter as input. In most areas of the globe soil porosity is between the 0.4 and 0.5 (Fig. 10). There are a few areas such as the northern Asia and northern North America where soil porosity is higher than 0.5 and there almost no areas where soil porosity is less than 0.35 (Fig. 10). In the future, if the soil porosity data will be corrected using *in-situ* clay, silt, and organic matter fractions, ASCAT SSM might be improved and might also lead to improvement in our 1^st^ and 2^nd^ merged SSM product.

### Errors derived from SMOS, FY3-B and SMAP SSM

Although the soil moisture retrieval algorithms of SMOS, FY3-B and SMAP SSM products are different, they are based on the same radiative transfer equation (Table 8), i.e. the zero-order radiative transfer *τ*-*w* model^60^. The difference between these soil moisture retrieval algorithms is that the applied parameters of the zero-order radiative transfer model to get best SSM retrieval are different. The disparities in brightness temperature, soil roughness model, vegetation optical depth, soil temperature, vegetation canopy temperature, vegetation single scattering albedo, soil dielectric model lead to the difference between SMOS, FY3-B and SMAP SSM^56^. These main inputs to the retrieval algorithms of SMOS, FY3-B and SMAP SSM also produce errors, which are transferred to our 1^st^ and 2^nd^ merged SSM data. Further detailed analysis of the error sources in the SMOS, FY3-B and SMAP SSM data would be helpful to improve the merged SSM data products described in this study.

**Table 8:** Main inputs of the retrieval algorithms of SMOS, FY3-B and SMAP SSM.

Foundational model	Zero-order radiative transfer model (i.e.,*τ*−*w* model)		
Algorithm	L-MEB for SMOS	Qp model for FY3-B	SCA-V for SMAP
Brightness temperature (TB)	1.4 GHz with muti-angles (2.5 ± 2.5 to 62.5 ± 2.5°)	10.65 and 36.5 GHz	Interpolated TB at 1.41GHz using the Backus-Gilbert method
Soil roughness model	QHN model: Q = 0; N_H-p_ = N_V-p_ = −1 (low vegetation); N_H-p_ = 1, N_V-p_ = −1 (V-p), (forest); H: from LUT based on IGBP (0.02 ≤ H ≤ 0.3)	Qp model: The coefficients of Qp model are estimated using simulated data from AIEM	QHN model: Q = 0; N = 2; H: from LUT based on IGBP (0.083 ≤ H ≤ 0.160)
Vegetation optical depth (*τ*)	*τ* is retrieved in L-MEB with soil moisture simultaneously	*τ* = b × VWC/cosθ: VWC: from an empirical function of LAI; θ: the incident angle.	*τ* = b × VWC: b: from LUT based on IGBP (0 ≤ b ≤ 0.13); VWC: from the nonlinear function of NDVI
Soil temperature (Ts)	from ECMWF (Level 1~3)	from an empirical linear function of TB at 36.5 GHz	from the GMAO-GEOS-FP (Level 1: 5~15cm and Level 2: 15–35 cm)
Canopy temperature (Tv)	from ECMWF (Level 1: top 0–7 cm)	Tv = Ts	Tv = Ts
Single scattering albedo (w)	from LUT based on IGBP (0.06 ≤ w ≤ 0.12)	w = 0	from LUT based on IGBP (0 ≤ w ≤ 0.07)
Soil dielectric model	Mironov *et al*.	/	Mironov *et al*.

*Q, H and N are the parameters of QHN model; H-p: Horizontal-polarization; V-p: Vertical-polarization; IGBP: the International Geosphere-Biosphere Programme; ECMWF: the European Center for Medium range Weather Forecasting; AIEM: the Advanced Integral Equation Method; VWC: the Vegetation Water Content; LAI: the Leaf Area Index; NDVI: the Normalized Difference Vegetation Index; GMAO-GEOS-FP: the Goddard Modelling and Assimilation Office-Goddard Earth Observing System (model)-Forward Processing; LUT: Look-Up-Table.

## Supplementary information

Supplementary validation

## Data Availability

The codes used for Global Daily-scale Soil Moisture Fusion Dataset (GDSMFD) are available in National Tibetan Plateau Data Center (10.11888/RemoteSen.tpdc.271988)^61^.
