# Supplementary material for: Global soil moisture data fusion by Triple Collocation Analysis from 2011 to 2018
Source: Sci Data. 2022 Nov 11;9:687. doi: 10.1038/s41597-022-01772-x (PMC9652308; doi:10.1038/s41597-022-01772-x)
Supplement: Supplementary file 1 — Supplementary validation [file 41597_2022_1772_MOESM1_ESM.docx]

**Supplementary validation**

**Contents**

[**1.** **Comparison with airborne observation-based soil moisture data.** 2](#_Toc116045026)

[**2.** **Comparison with other fusion soil moisture products.** 3](#_Toc116045027)

[**References** 5](#_Toc116045028)

1. **Comparison with airborne observation-based soil moisture data.**

Airborne observation-based soil moisture data with a gird resolution of 1 km in the Shandian river basin of Inner Mongolia in September 2018 covering areas of 70 km× 12 km were used as the reference of true soil moisture to evaluate the satellite SSM products and the merged SSM developed by this study using the TCA-LWF algorithm. Airborne observation-based soil moisture data used the SCA-V (Single Channel Algorithm-Vertical polarization) soil moisture retrieval algorithm same with SMAP product (Fig.1)^1^.


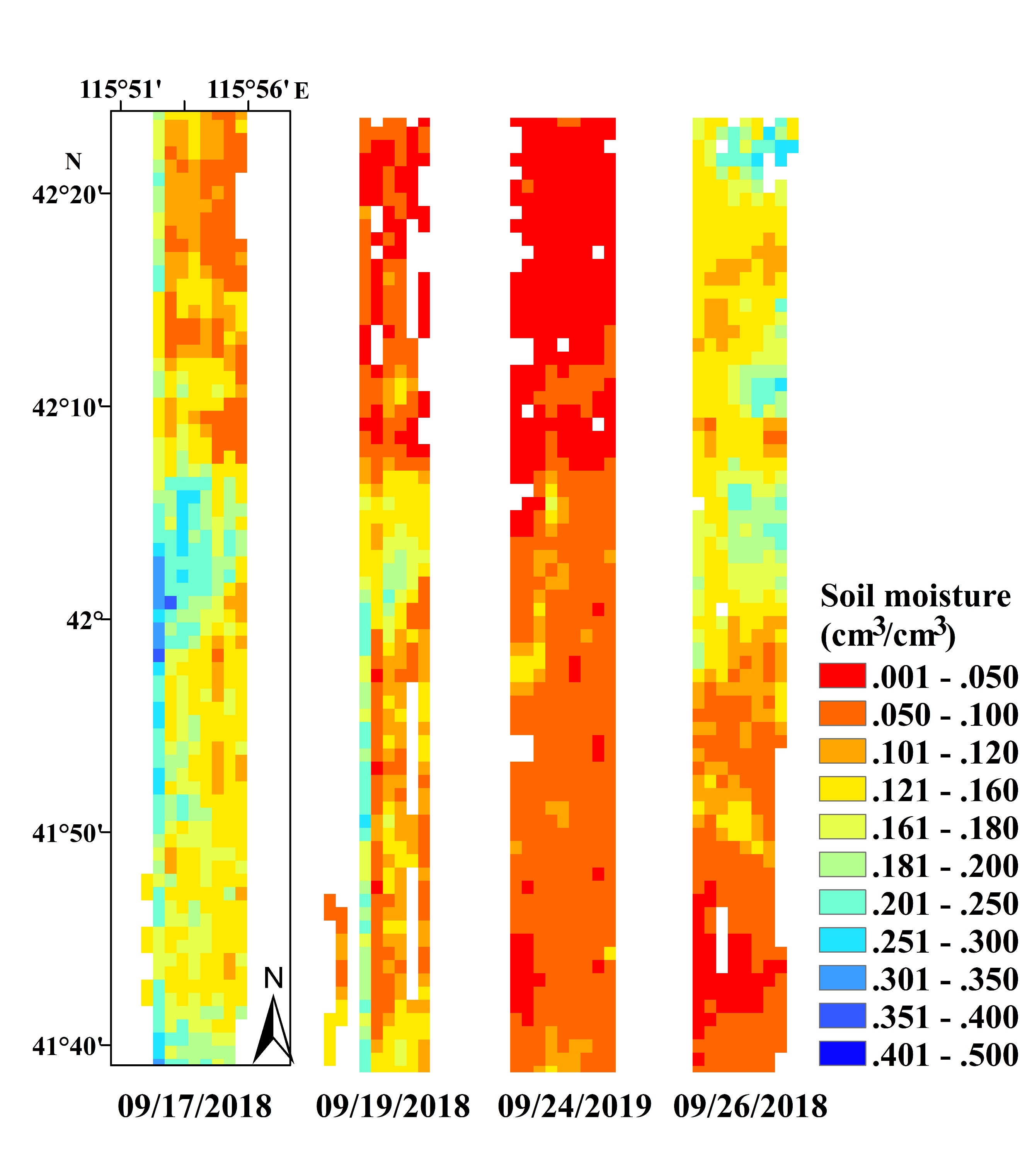


Figure 1 Airborne observation-based soil moisture data on September 17, 19, 24 and 26, 2018 in Shandian river basin of Inner Mongolia

We calculated the values of MAE (Mean Absolute Error), RMSE (Root Mean Square Error), RB (Relative Bias) and *R* (Correlation Coefficient) between the airborne observation-based soil moisture and the satellite/merged SSM products on September 17, 19, 24 and 26, 2018 respectively (Table 1).

From Table 1, there were not soil moisture values for SMOS product in the Shandian river basin of Inner Mongolia on September 17, 19, 24 and 26, 2018. The best soil moisture product with consistent with the airborne radiometer observation-based soil moisture was SMAP product, and the MAE (0.040 cm^3^/cm^3^), RMSE (0.045 cm^3^/cm^3^) and RB (0.159) values of SMAP were least than FY3-B, ASCAT, ESA-CCI, 1^st^ merged and 2^nd^ merged soil moisture products. One of the reasons was maybe that the airborne radiometer observation-based soil moisture data adopted the same soil moisture retrieval algorithm with SMAP product i.e., SCA-V and referred to the same parameter values of SCA-V. Except SMAP product, 2^nd^ merged soil moisture product had better performance in term of consistence with the airborne observation-based soil moisture data than FY3-B, ASCAT, ESA-CCI and 1^st^ merged soil moisture products.

Table 1 MAE (cm^3^/cm^3^), RMSE (cm^3^/cm^3^), RB and *R* values of SMOS, FY3-B, ASCAT, ESA-CCI, SMAP, the 1^st^ and 2^nd^ merged SSM against airborne observation-based soil moisture.

| Indies | SMOS | FY3-B | ASCAT | 1^st^ merged SSM | ESA-CCI | SMAP | 2^nd^ merged SSM |
| --- | --- | --- | --- | --- | --- | --- | --- |
| MAE | / | 0.078 | 0.137 | 0.117 | 0.226 | 0.040 | 0.075 |
| RMSE | / | 0.088 | 0.145 | .0119 | 0.094 | 0.045 | 0.082 |
| RB | / | 0.613 | 1.167 | -1.068 | 0.775 | 0.159 | -0.687 |
| *R* | / | 0.588 | 0.588 | 0.751 | 0.575 | 0.735 | 0.878 |

1. **Comparison with other fusion soil moisture products.**

Two merged global soil moisture products using the neural network fusion algorithm were used to compare with our merged soil moisture product (Global Daily-scale Soil Moisture Fusion Dataset, GDSMFD). These two soil moisture products were the Yao’s global daily surface soil moisture dataset (named as NNsm) with a spatial resolution of 36 km from June 2002 to December 2019, and the Chen’s remote-sensing-based surface soil moisture dataset (named as RSSSM) covering 2003–2018 at 0.1◦ of spatial resolution and 10-day of temporal resolution^2,3^.

To maintain consistent temporal resolution, the GDSMFD and Yao’s NNsm products were sampled to the 10-day temporal resolution same with RSSSM product. And, we analysed the spatial difference between three fusion soil moisture products of GDSMFD, NNsm and RSSSM, and compared the spatial distribution of three fusion soil moisture products with fractional of vegetation cover (Fig.2). Vegetation coverage data of GLASS (Global Land Surface Satellite) that was released by National Earth System Science Data Center, National Science & Technology Infrastructure of China (<http://www.geodata.cn>) was used in this study^4,5^. This vegetation coverage data has a spatial grid resolution of 0.5°and a temporal resolution of 8-day covering global area. Therefore, this vegetation data was resampled 25km of spatial grid resolution by interpolation of adjacent pixels to keep consistent spatial grid resolution (i.e., 25km) with our fusion soil moisture product i.e., GDSMFD.

Comparing with the NNsm dataset, the GDSMFD has higher spatial grid resolution (25km) than 36km. Comparing with the RSSSM dataset, the GDSMFD has higher temporal resolution(daily) than 10 days. From Fig.2, the spatial distribution pattern of GDSMFD has basically consistent spatial pattern with vegetation coverage data in global scale, and also is same with NNsm and RSSSM products except some high vegetation coverage areas such as the northern areas of Asia, the Amazon rainforest region of South America and the Congo Basin rainforest region of Africa. The big difference between the GDSMFD, NNsm and RSSSM is mainly concentrated in the high vegetation coverage areas (≥0.7) i.e., the tree areas. In the tree areas, the soil moisture values of NNsm and RSSSM products are obviously higher than soil moisture values of GDSMFD.


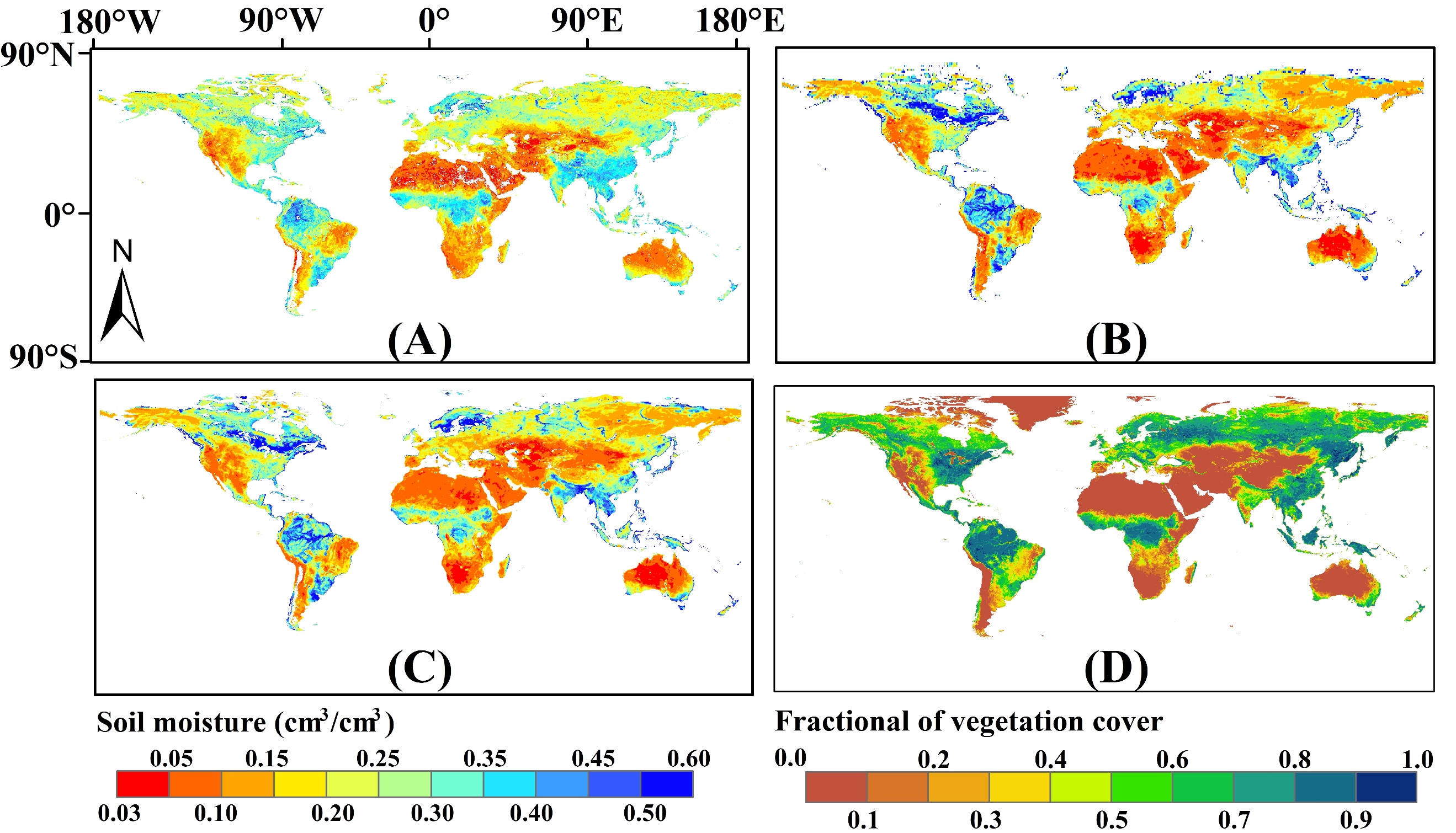


Figure 2 Spatial patters of mean soil moisture value three fusion soil moisture products from August 1 to 10, 2015 i.e., (A) GDSMFD; (B) NNsm; (C) RSSSM, and mean vegetation coverage from August 5 to 12, 2015 i.e., (D)

**References**

1. Zhao, T. *et al.* Soil moisture experiment in the Luan River supporting new satellite mission opportunities. *Remote Sens. Environ.* **240**, 111680, https://doi.org/https://doi.org/10.1016/j.rse.2020.111680 (2020).

2. Yao, P. *et al.* A long term global daily soil moisture dataset derived from AMSR-E and AMSR2 (2002–2019). *Sci. Data* **8**, 1–17, https://doi.org/10.1038/s41597-021-00925-8 (2021).

3. Chen, Y., Feng, X. & Fu, B. An improved global remote-sensing-based surface soil moisture (RSSSM) dataset covering 2003-2018. *Earth Syst. Sci. Data* **13**, 1–31, https://doi.org/10.5194/essd-13-1-2021 (2021).

4. Liang, S. *et al.* The global land surface satellite (GLASS) product suite. *Bull. Am. Meteorol. Soc.* **102**, https://doi.org/10.1175/BAMS-D-18-0341.1 (2021).

5. Jia, K. *et al.* Global Land Surface Fractional Vegetation Cover Estimation Using General Regression Neural Networks From MODIS Surface Reflectanc. **53**, 4787–4796, https://doi.org/10.1109/TGRS.2015.2409563 (2015).
